# Some novel soliton solution, breather solution and Darboux transformation for a generalized coupled Toda soliton hierarchy

**DOI:** 10.1038/s41598-018-33212-5

**Published:** 2018-10-16

**Authors:** Fajun Yu, Li Li, Shuo Feng

**Affiliations:** School of Mathematics and Systematic Sciences, Shenyang Normal University, Shenyang, 110034 China

## Abstract

A few of discrete integrable coupling systems(DICSs) of previous papers are linear discrete integrable couplings(LDICS). We take a special matrix Lie algebra system(non-semisimple) to construct the Lax pairs, and establish a method for deriving the nonlinear discrete integrable coupling systems(NDICS). From the Lax pairs of the generalized Toda(G-Toda) spectral problem, we can derive a novel NDICS, which is a real NDICS. For the obtained lattice integrable coupling equation, we establish a Darboux transformation (DT) with 4 × 4 Lax pairs, and apply the gauge transformation to a specific equation, then the explicit solutions of the lattice integrable coupling equation are given, which contains discrete soliton solution, breather solution and rogue wave solution. Furthermore, we can derive the discrete explicit solutions with free parameters to depict their dynamic behaviors.

## Introduction

A few of discrete integrable equations can describe some important physical phenomena, which are the focus of common concern in fields of mathematical physics. There is an interesting work to search for a novel integrable discrete equation. The discrete lattice systems not only have rich mathematical structures but also have many applications in mathematical physics, including the mathematical physics, numerical analysis, statistical physics, quantum physics^1–4^, and so on. A few of integrable equations have some important soliton solutions including both continuous and discrete, which are exponentially decaying at spatial infinity. Especially, the Toda lattice equation is one of the well-known lattice equations, which can exhibit the soliton phenomenon^5^. The equations of motion of Toda’s exponential lattice were completely discovered analytical expressions for the constants of the motion with computer^6^. A systematic procedure was considered to establish an infinite series of local conserved densities in^7^.

Ma and Fuchsstiener firstly considered a kind of interesting integrable couplings(ICS) in^1,2^. Those obtained integrable couplings have abundant mathematical structures, then some new methods to search for an integrable couplings are constructed. For examples, Ma present the perturbation method to establish an integrable couplings in refs^8,9^. Zhang, Xia and Fan construct some novel ICSs with the extend Lie algebra method in^10–15^. The discrete integrable couplings are proposed through the Lie algebras(semi-direct sums form)^16^, and which is a beautiful method and has Hamiltonian structures^17^. Furthermore, the component-trace identity is constructed to solve the Hamiltonian structure of some discrete ICSs by Ma and Zhang in^18^. And the other methods are presented to construct the Hamiltonian structure of discrete integrable system, such as a discrete variational identity is considered by Lie algebra of semi-direct sums form in^19^, three kinds of coupling ICSs of the KdV soliton equations are studied through the four Lie algebras in *R*^6^ ^20–23^. Over the previous work, the most discrete ICSs are LDICs. However, the NDICs are more important in applications. The nonlinear continuous an discrete integrable Hamiltonian couplings are obtained by using the non-semisimple Lax pairs in refs^24,25^. Yu consider a real continuous nonlinear ICS and its Hamiltonian structure in^26^.

Some nonlinear phenomena can be described by partial differential equations in nature, and some models are the infinite dimensional integrable systems. To search for the solutions of the partial differential equations, many novel methods are presented in refs^27–29^, such as Darboux transformation(DT)^30^, inverse scattering transformation^31^, Bäcklund transformation (BT)^32–34^ and Hirota method^35^. There are some classical methods to obtain the DT for partial differential equations, such as the operator decomposition^36^, gauge transformation method^37,38^, loop group technics^39^ and Riemann-Hilbert method^40^ are proposed to solve the soliton solutions of partial differential equations. Some multi-soliton and localized solutions of integrable equations are considered in different dimensions integrable equations^41–44^ by DT method. Some ICSs of triangular system are preposed through the DT method of Lax pair in^45^.

In this paper, we extend the previous method to a new nonlinear discrete integrable couplings. A method for constructing NDICS is presented with special non-semisimple matrix Lax pairs. A novel NDICS is given by a direct application to the generalized Toda spectral problem, which is normally nonlinear discrete integrable couplings. Furthermore, the explicit solutions of the soliton equation are received by the Darboux transformation with 4 × 4 Lax pairs. We take using the Darboux transformation to the discrete coupling soliton equations and derive some novel discrete explicit solutions, which is an important and formidable task in soliton theory.

## Results

### A nonlinear integrable couplings

The ICS is proposed through Virasoro symmetry algebra^8^. The

1 $${u}_{nt}=K({u}_{n}),$$

is a famous integrable system, the next system

2 $$\begin{array}{rcl}{u}_{nt} & = & K({u}_{n}),\\ {v}_{nt} & = & S({u}_{n},{v}_{n}),\end{array}$$

is called a discrete ICS of the system (1), if ${v}_{nt}=S({u}_{n},{v}_{n})$ is also integrable and $S({u}_{n},{v}_{n})$ contains explicitly *u*_*n*_ or *u*_*n*_-derivatives with respect to shift operator *E*. In such ICS, the supplementary variable *v*_*n*_ is linear with respect to *v*_*n*_.

If the second equation of a discrete ICS

3 $${u}_{nt}=K({u}_{n}),\,{v}_{nt}=S({u}_{n},{v}_{n}),$$

is a nonlinear equation for *v*_*n*_, then the whole system can be called a NDICS of ${u}_{nt}=K({u}_{n})$.

In this paper, we focus on how to construct the integrable partial differential equations and concern with a natural question: How can one construct nonlinear discrete integrable couplings? We take a kind of non-semisimple Lie algebra:

$$\bar{g}=(\begin{array}{cc}A & B\\ 0 & A+B\end{array}),\,A,B\in g.$$

The spectral matrices are shown as following:

5 $$\bar{U}=(\begin{array}{cc}U({u}_{n}) & {U}_{a}({v}_{n})\\ 0 & U({u}_{n})+{U}_{a}({v}_{n})\end{array})$$

6 $$\bar{V}=(\begin{array}{cc}V({u}_{n}) & {V}_{a}({v}_{n})\\ 0 & V({u}_{n})+{V}_{a}({v}_{n})\end{array})$$

which can engender a NDICS. All matrices above are closed under the matrix product, and which constitute the matrix Lie under the matrix commutator. The non-semisimple Lie algebras have a non-trivial ideal Lie sub-algebra consisting of matrices of the following form

7 $$(\begin{array}{cc}0 & {U}_{a}\\ 0 & {U}_{a}\end{array}).$$

Based on the Lax pair

8 $$E{\rm{\Phi }}=\bar{U}{\rm{\Phi }},\,{{\rm{\Phi }}}_{t}=\bar{V}{\rm{\Phi }},$$

and the compatibility of the enlarged discrete zero curvature equation

9 $${\bar{U}}_{t}=(E\bar{V})\bar{U}-\bar{U}\bar{V}$$

we yield the following system

10 $$\{\begin{array}{rcl}{U}_{t} & = & (EV)U-UV,\\ {U}_{a,t} & = & (E{V}_{a})U-U{V}_{a}+(EV){U}_{a}-{U}_{a}V+(E{V}_{a}){U}_{a}-{U}_{a}{V}_{a}.\end{array}$$

This is a really nonlinear ICS, since the matrix $(E{V}_{a}){U}_{a}-{U}_{a}{V}_{a}$ can produce some nonlinear terms.

In order to construct a NDICS of soliton hierarchy, we use the Lie algebra *G* and present the block matrix of Lie algebras $\bar{G}$. We next shed light on the general idea to construct the ICS by a block type matrix of Lie algebra:11

We take a novel Lax pairs of block type matrix:12substituting a pair matrix spectral (12) into a stationary zero curvature equation

13 $$(E\bar{V})\bar{U}-\bar{U}\bar{V}=0,$$

a set of novel stationary curvature equations are presented as following

14 $$\{\begin{array}{l}(E{V}_{0}){U}_{0}-{U}_{0}{V}_{0}=0,\\ (E{V}_{1}){U}_{1}-{U}_{1}{V}_{1}+(E{V}_{1}){U}_{0}-{U}_{0}{V}_{1}+(E{V}_{0}){U}_{1}-{U}_{1}{V}_{0}=0,\\ (E{V}_{2}){U}_{2}-{U}_{2}{V}_{2}+(E{V}_{1}){U}_{1}-{U}_{1}{V}_{1}+(E{V}_{2}){U}_{0}-{U}_{0}{V}_{2}+(E{V}_{0}){U}_{2}-{U}_{2}{V}_{0}=0,\end{array}$$

and a new form of discrete zero curvature equation is obtained

15 $$\{\begin{array}{rcl}{U}_{0t} & = & (E{V}_{0}){U}_{0}-{U}_{0}{V}_{0},\\ {U}_{1t} & = & (E{V}_{1}){U}_{1}-{U}_{1}{V}_{1}+(E{V}_{1}){U}_{0}-{U}_{0}{V}_{1}+(E{V}_{0}){U}_{1}-{U}_{1}{V}_{0},\\ {U}_{2t} & = & (E{V}_{2}){U}_{2}-{U}_{2}{V}_{2}+(E{V}_{1}){U}_{1}-{U}_{1}{V}_{1}+(E{V}_{2}){U}_{0}-{U}_{0}{V}_{2}+(E{V}_{0}){U}_{2}-{U}_{2}{V}_{0}.\end{array}$$

The first equation of Eq. (15) is similar to the Eq. (3), the Eq. (15) provides normally a NDICS for Eq. (3). So, the block type matrix of $\bar{G}$ is a novel Lie algebra and it provides a novel choice of candidates of discrete ICS for Eq. (15) with G.

In this section, we obtain a set of new Lie algebras, which can generate a NDICS. More specifically, we show the choice of spectral matrices as following:

16 $$\bar{U}=(\begin{array}{cc}U({u}_{n}) & {U}_{a}({v}_{n})\\ 0 & U({u}_{n})+{U}_{a}({v}_{n})\end{array})$$

which can be derived a NDICS.

### A real NDICS of the generalized Toda lattice hierarchy

Based on the Lie algebra theory, some discrete ICSs of the known equation hierarchies have been presented, such as the Toda hierarchy, the modified KdV lattice equation, Volterra lattice equation *et al*.^10,11,16^. We will illustrate a novel method to construct a real NDICS by the non-semisimple algebra.

We present a novel Lax pairs $\bar{U}$ and $\bar{V}$, which is as following

17 $$\begin{array}{rcl}\bar{U}({\bar{u}}_{n}) & = & (\begin{array}{cc}U({u}_{n}) & {U}_{a}({v}_{n})\\ 0 & U({u}_{n})+{U}_{a}({v}_{n})\end{array}),\\ \bar{V}({\bar{u}}_{n}) & = & (\begin{array}{cc}V({u}_{n}) & {V}_{a}({\bar{u}}_{n})\\ 0 & V({u}_{n})+{V}_{a}({\bar{u}}_{n})\end{array}),\end{array}$$

with

18 $$\begin{array}{ll}U=(\begin{array}{cc}0 & -{r}_{n}\\ {q}_{n} & \lambda \end{array}), & {U}_{a}(v)=(\begin{array}{cc}0 & {v}_{n}\\ {w}_{n} & 0\end{array}),\\ V=(\begin{array}{cc}{a}_{n} & {b}_{n}\\ {c}_{n} & -{a}_{n}\end{array}), & {V}_{a}({u}_{n},{v}_{n},\lambda )=(\begin{array}{cc}{e}_{n} & {f}_{n}\\ {g}_{n} & -{e}_{n}\end{array}).\end{array}$$

Obviously, we have the following system

19 $$\begin{array}{rcl}(EV)U-UV & = & (\begin{array}{cc}{q}_{n}{b}_{n+1}+{r}_{n}{c}_{n} & -{r}_{n}({a}_{n}+{a}_{n+1})+{b}_{n+1}\lambda \\ {q}_{n}({a}_{n}+{a}_{n+1})+{c}_{n}\lambda  & -{r}_{n}{c}_{n+1}-{q}_{n}{b}_{n}+({a}_{n}-{a}_{n+1})\lambda \end{array}),\\ (E{V}_{a})U-U{V}_{a} & = & (\begin{array}{cc}{q}_{n}\,{f}_{n+1}+{r}_{n}\,{g}_{n} & -{r}_{n}({e}_{n+1}+{e}_{n})+\lambda {f}_{n}\\ -{q}_{n}({e}_{n+1}+{e}_{n})-\lambda {g}_{n} & -{r}_{n}\,{g}_{n+1}-{q}_{n}\,{f}_{n}-\lambda {e}_{n+1}+\lambda {e}_{n}\end{array}),\\ EV){U}_{a}-{U}_{a}V & = & (\begin{array}{cc}{w}_{n}{b}_{n+1}-{v}_{n}{c}_{n} & {v}_{n}({a}_{n+1}+{a}_{n})\\ -{w}_{n}({a}_{n+1}+{a}_{n}) & -{w}_{n}{b}_{n}+{v}_{n}{c}_{n+1}\end{array}),\\ (E{V}_{a}){U}_{a}-{U}_{a}{V}_{a} & = & (\begin{array}{cc}{w}_{n}\,{f}_{n+1}-{v}_{n}\,{g}_{n} & {v}_{n}({e}_{n+1}+{e}_{n})\\ -{w}_{n}({e}_{n+1}+{e}_{n}) & {v}_{n}\,{g}_{n+1}-{w}_{n}\,{f}_{n}\end{array}).\end{array}$$

Based on the stationary discrete zero curvature equation

20 $$\{\begin{array}{l}(EV)U-UV=0,\\ (E{V}_{a})U-U{V}_{a}+(EV){U}_{a}-{U}_{a}V+(E{V}_{a}){U}_{a}-{U}_{a}{V}_{a}=0,\end{array}$$

it gives rise to

21 $$\begin{array}{l}{q}_{n}{b}_{n+1}+{r}_{n}{c}_{n}=0,\\ -{r}_{n}({a}_{n}+{a}_{n+1})+{b}_{n+1}\lambda =0,\\ {q}_{n}({a}_{n}+{a}_{n+1})+{c}_{n}\lambda =0,\\ -{r}_{n}{c}_{n+1}-{q}_{n}{b}_{n}+({a}_{n}-{a}_{n+1})\lambda =0,\\ {q}_{n}\,{f}_{n+1}+{r}_{n}\,{g}_{n}+{w}_{n}{b}_{n+1}-{v}_{n}{c}_{n}+{w}_{n}\,{f}_{n+1}-{v}_{n}\,{g}_{n}=0,\\ {v}_{n}({a}_{n+1}+{a}_{n})-{r}_{n}({e}_{n+1}+{e}_{n})+\lambda {f}_{n}+{v}_{n}({e}_{n+1}+{e}_{n})=0,\\ -{q}_{n}({e}_{n+1}+{e}_{n})-\lambda {g}_{n}-{w}_{n}({a}_{n+1}+{a}_{n})-{w}_{n}({e}_{n+1}+{e}_{n})=0,\\ -{r}_{n}\,{g}_{n+1}-{q}_{n}\,{f}_{n}-\lambda {e}_{n+1}+\lambda {e}_{n}\\ -{w}_{n}{b}_{n}+{v}_{n}{c}_{n+1}+{v}_{n}\,{g}_{n+1}-{w}_{n}\,{f}_{n}=0.\end{array}$$

Setting

$$a=\sum _{m\ge 0}\,{a}^{m}{\lambda }^{-m},\,b=\sum _{m\ge 0}\,{b}^{m}{\lambda }^{-m},\,c=\sum _{m\ge 0}\,{c}^{m}{\lambda }^{-m},$$

and

$$e=\sum _{m\ge 0}\,{e}_{m}{\lambda }^{-m},\,f=\sum _{m\ge 0}\,{f}_{m}{\lambda }^{-m},\,g=\sum _{m\ge 0}\,{g}_{m}{\lambda }^{-m}.$$

Taking the above some equations into the stationary discrete zero curvature equation (20), we can get

22 $$\{\begin{array}{l}{q}_{n}{b}_{n+1}^{(m)}+{r}_{n}{c}_{n}^{(m)}=0,\\ {b}_{n+1}^{(m+1)}-{r}_{n}({a}_{n}^{(m)}+{a}_{n+1}^{(m)})=0,\\ {c}_{n}^{(m+1)}+{q}_{n}({a}_{n}^{(m)}+{a}_{n+1}^{(m)})=0,\\ ({a}_{n}^{(m+1)}-{a}_{n+1}^{(m+1)})-{r}_{n}{c}_{n+1}^{m}-{q}_{n}{b}_{n}^{(m)}=0,\\ {q}_{n}\,{f}_{n+1}^{(m)}+{r}_{n}\,{g}_{n}^{(m)}+{w}_{n}{b}_{n+1}^{(m)}-{v}_{n}{c}_{n}^{(m)}+{w}_{n}\,{f}_{n+1}^{(m)}-{v}_{n}\,{g}_{n}^{(m)}=0,\\ {v}_{n}({a}_{n+1}^{(m)}+{a}_{n}^{(m)})-{r}_{n}({e}_{n+1}^{(m)}+{e}_{n}^{(m)})+{f}_{n}^{(m+1)}+{v}_{n}({e}_{n+1}^{(m)}+{e}_{n}^{(m)})=0,\\ -{q}_{n}({e}_{n+1}^{(m)}+{e}_{n}^{(m)})-{g}_{n}^{(m+1)}-{w}_{n}({a}_{n+1}^{(m)}+{a}_{n}^{(m)})-{w}_{n}({e}_{n+1}^{(m)}+{e}_{n}^{(m)})=0,\\ -{r}_{n}\,{g}_{n+1}^{(m)}-{q}_{n}\,{f}_{n}^{(m)}-{e}_{n+1}^{(m+1)}+{e}_{n}^{(m+1)}-{w}_{n}{b}_{n}^{(m)}+{v}_{n}{c}_{n+1}^{(m)}+{v}_{n}\,{g}_{n+1}^{(m)}-{w}_{n}\,{f}_{n}^{(m)}=0.\end{array}$$

Setting ${a}_{n}^{(0)}=-\,\frac{1}{2}$, ${f}_{n}^{(0)}={g}_{n}^{(0)}={e}_{n}^{(0)}=0$, a few series are given as following

23 $$\{\begin{array}{l}{b}_{n}^{(0)}=0,\,{c}_{n}^{(0)}=0,\\ {a}_{n+1}^{(1)}-{a}_{n}^{(1)}=-\,{r}_{n}{c}_{n+1}^{(0)}-{q}_{n}{b}_{n}^{(0)},\\ {a}_{n}^{(1)}=-\,{r}_{n-1}{q}_{n},\,{b}_{n}^{(1)}=-\,{r}_{n-1},\,{c}_{n}^{(1)}={q}_{n},\\ {f}_{n}^{(1)}={v}_{n},\,{g}_{n}^{(1)}={w}_{n},\,{e}_{n}^{(1)}=0,\\ {f}_{n}^{(2)}={q}_{n+1}{r}_{n}{v}_{n}+{q}_{n}{r}_{n-1}{v}_{n},\\ {g}_{n}^{(2)}={q}_{n}{r}_{n-1}{w}_{n}+{q}_{n+1}{r}_{n}{w}_{n},\\ (E-1){e}_{n}^{2}={v}_{n}({w}_{n+1}-{w}_{n})+{v}_{n}({q}_{n+1}-{q}_{n})+{w}_{n}{r}_{n-1}-{w}_{n+1}{r}_{n}.\end{array}$$

Letting

$${{\rm{\Delta }}}_{n}^{(m)}=(\begin{array}{cc}-2{a}_{n}^{(m)} & 0\\ 0 & 0\end{array}),\,{\tilde{V}}_{n}^{(m)}={V}_{n}^{(m)}+{{\rm{\Delta }}}_{n},$$

and

24 $${{\rm{\Delta }}}_{an}^{(m)}=(\begin{array}{cc}0 & 0\\ 0 & {e}_{n}^{(m+1)}\end{array}),\,{\tilde{V}}_{an}^{(m)}={V}_{an}^{(m)}+{{\rm{\Delta }}}_{an},$$

we get

$$(E{\tilde{V}}_{n}^{(m)})U-U{\tilde{V}}_{n}^{(m)}=(\begin{array}{cc}0 & -{b}_{n+1}^{(m+1)}+2{r}_{n}{a}_{n+1}^{(m)}\\ {c}_{n}^{(m+1)}+2{q}_{n}{a}_{n}^{(m)} & {a}_{n+1}^{(m+1)}-{a}_{n}^{(m+1)}\end{array}),\,m\ge 0,$$

and

25 $$\begin{array}{l}(E{\tilde{V}}_{a})U-U{\tilde{V}}_{a}+(E\tilde{V}){U}_{a}-{U}_{a}\tilde{V}+(E{\tilde{V}}_{a}){U}_{a}-{U}_{a}{\tilde{V}}_{a}\\ \,=\,(\begin{array}{cc}0 & {f}_{n}^{(m+1)}+2{v}_{n}{a}_{n+1}^{(m)}-{v}_{n}({e}_{n+1}^{(m+1)}+{e}_{n}^{(m+1)})\\ -{g}_{n}^{(m+1)}-2{w}_{n}{a}_{n}^{(m)}+{w}_{n}({e}_{n+1}^{(m+1)}+{e}_{n}^{(m+1)}) & 0\end{array}),\end{array}$$

where $$m\ge 0$$.

The nonlinear lattice equation hierarchy is derived through the Tu method and the discrete zero equation (10) as following

26 $${\bar{U}}_{tn}={(\begin{array}{c}{r}_{n}\\ {q}_{n}\\ {v}_{n}\\ {w}_{n}\end{array})}_{t}=(\begin{array}{c}-{b}_{n+1}^{(m+1)}+2{r}_{n}{a}_{n+1}^{(m)}\\ {c}_{n}^{(m+1)}+2{q}_{n}{a}_{n}^{(m)}\\ {f}_{n}^{(m+1)}+2{v}_{n}{a}_{n+1}^{(m)}-{v}_{n}({e}_{n+1}^{(m+1)}+{e}_{n}^{(m+1)})\\ -{g}_{n}^{(m+1)}-2{w}_{n}{a}_{n}^{(m)}+{w}_{n}({e}_{n+1}^{(m+1)}+{e}_{n}^{(m+1)})\end{array}).$$

According to the Eqs (23) and (26), the 1-nd system in (26) is derived, when *m* = 1,

27 $$\{\begin{array}{rcl}{r}_{n,{t}_{1}} & = & {r}_{n}({q}_{n}{r}_{n-1}-{q}_{n+1}{r}_{n}),\\ {q}_{n,{t}_{1}} & = & {q}_{n}({q}_{n+1}{r}_{n}-{q}_{n}{r}_{n-1}),\\ {v}_{n,{t}_{1}} & = & {v}_{n}({q}_{n}{r}_{n-1}-{q}_{n+1}{r}_{n})-{v}_{n}^{2}[({w}_{n+1}-{w}_{n})+({q}_{n+1}-{q}_{n})]{v}_{n}{w}_{n}{r}_{n-1}+{v}_{n}{w}_{n+1}{r}_{n},\\ {w}_{n,{t}_{1}} & = & -{w}_{n}({q}_{n}{r}_{n-1}-{q}_{n+1}{r}_{n})+{v}_{n}{w}_{n}[({w}_{n+1}-{w}_{n})+({q}_{n+1}-{q}_{n})]+{w}_{n}{w}_{n}{r}_{n-1}-{w}_{n}{w}_{n+1}{r}_{n}.\end{array}$$

We obtain the NDICS of a G-Toda lattice equation (27) with a non-semisimple algebra, and it is normally a NDICS because the matrix $(E{V}_{a}){U}_{a}-{U}_{a}{V}_{a}$ produces the nonlinear terms. The nonlinear discrete equations (NDEs) can describe many phenomena in physics, chemistry, and biology. Many NDEs are proposed and investigated, such as the Toda lattice equation, the Volterra lattice equation, the discrete nonlinear Schrödinger equation and so on. A lot of analytical methods are presented to study NDEs from different points of view from integrability to chaos. The celebrated Toda lattice is introduced by Toda by considering the lattice with exponential interaction^46^ and thereafter many applications of the Toda lattice have been studied, thus promoting the developments of integrable systems, random matrix theory, conformal field theory^47^ and so on.

The system is first derived as a model equation for the numerical simulation of the complex modified Korteweg-de Vries equation ${v}_{t}={v}_{xxx}+6|v{|}^{2}{v}_{x}$ which is a prototypical integrable partial differential equation for a wide range of physical phenomena, the propagation of few-cycle optical pulses in cubic nonlinear media^48^, the transmission of electromagnetic waves in nematic waveguides^49^, the propagation of transverse waves in a molecular chain model^50^, and so on.

If Eq. (27) has two integrals of motion:

28 $${r}_{n}{q}_{n}=D(n),\,{v}_{n}{w}_{n}=C(n),$$

where *D*(*n*) and *C*(*n*) are the arbitrary functions, we can obtain the physical meaning of these equations through investigating in the continual limit: ${f}_{n\pm 1}=f\pm \frac{\partial f}{\partial n}+\frac{1}{2}\frac{{\partial }^{2}f}{\partial {n}^{2}}\pm \ldots$. And, the system (27) looks like approximately:

29 $$\frac{\partial r}{\partial t}\cong -\,\frac{dD}{dn}r+\frac{\partial }{\partial n}(D\frac{\partial r}{\partial n})-\frac{D}{r}(\frac{\partial r}{\partial n}),\,q=\frac{D}{r},$$

and

30 $$\frac{\partial v}{\partial t}+C(1+\frac{r}{v})\frac{\partial v}{\partial n}\cong -\,\frac{d}{dn}(D+C)v-\frac{\partial q}{\partial n}{v}^{2}+2\frac{dC}{dn}r+C\frac{\partial r}{\partial n},\,w=\frac{C}{v}.$$

Therefore, we obtain that the set of equation (27) describes the nonlinear diffusion wave processes in the dissipative discrete medium, the *D*(*n*) and *C*(*n*) are the diffusion coefficient and the wave velocity respectively. The nonlinear diffusion equation can describe some phenomena, such as the kinetics of phase transitions^51^, combined effects of population growth and diffusion^52^, propagation of signals in electric circuits^53^.

### Some novel soliton solution, breather solution and Darboux Transformation

The matrix $\tilde{{U}_{n}}$ defined has the same form as *U*_*n*_, that is,

31 $${\tilde{U}}_{n}=(\begin{array}{cccc}0 & -{\tilde{r}}_{n} & 0 & {\tilde{v}}_{n}\\ {\tilde{q}}_{n} & \lambda  & {\tilde{w}}_{n} & 0\\ 0 & 0 & 0 & {\tilde{v}}_{n}-{\tilde{r}}_{n}\\ 0 & 0 & {\tilde{q}}_{n}+{\tilde{w}}_{n} & \lambda \end{array}),$$

the novel transformation relations between old and new potentials are defined as following

32 $$\{\begin{array}{rcl}{\tilde{r}}_{n} & = & -{B}_{n+1}^{N-1}+{r}_{n},\\ {\tilde{q}}_{n} & = & -{C}_{n}^{N-1}+{q}_{n},\\ 2{\tilde{v}}_{n} & = & {G}_{n+1}^{N-1}-{B}_{n+1}^{N-1}+2{v}_{n},\\ 2{\tilde{w}}_{n} & = & {C}_{n}^{N-1}-{H}_{n}^{N-1}+2{w}_{n}.\end{array}$$

In this section, we will apply the DT to construct exact solutions of Eq. (27). Firstly, we consider a set of seed solutions (−1, 0, 1, 0) of Eq. (27), which are ${r}_{n}=-\,1,\,{q}_{n}=0,\,{\nu }_{n}=1,\,{\omega }_{n}=0$. Substituting the solutions into Eqs (17) and (18), we have the relations as following

33 $$E{\phi }_{n}=(\begin{array}{cccc}0 & 1 & 0 & 1\\ 0 & \lambda  & 0 & 0\\ 0 & 0 & 0 & 2\\ 0 & 0 & 0 & \lambda \end{array})\,{\phi }_{n},$$

and

34 $${\phi }_{{n}_{t}}=(\begin{array}{cccc}-\frac{\lambda }{2} & 1 & 0 & 1\\ 0 & \frac{\lambda }{2} & 0 & 0\\ 0 & 0 & -\frac{\lambda }{2} & 2\\ 0 & 0 & 0 & \frac{\lambda }{2}\end{array}).$$

After some algebra calculating of Eqs (33) and (34), we have the following two linear independent solutions ${\phi }_{n}={({\phi }_{n}^{1},{\phi }_{n}^{2},{\phi }_{n}^{3},{\phi }_{n}^{4})}^{T}$, ${\psi }_{n}={({\psi }_{n}^{1},{\psi }_{n}^{2},{\psi }_{n}^{3},{\psi }_{n}^{4})}^{T}$, in which the representations are obtained as following

35 $$\begin{array}{l}\{\begin{array}{rcl}{\phi }_{n}^{1} & = & 0,\\ {\phi }_{n}^{2} & = & -\,{\lambda }^{n+1}exp(\frac{\lambda }{2}t),\\ {\phi }_{n}^{3} & = & 2{\lambda }^{n}exp(\frac{\lambda }{2}t),\\ {\phi }_{n}^{4} & = & {\lambda }^{n+1}exp(\frac{\lambda }{2}t),\end{array}\end{array}$$

and

36 $$\{\begin{array}{rcl}{\psi }_{n}^{1} & = & {\lambda }^{n}exp(\frac{\lambda }{2}t),\\ {\psi }_{n}^{2} & = & -\,{\lambda }^{n+1}exp(\frac{\lambda }{2}t),\\ {\psi }_{n}^{3} & = & 4{\lambda }^{n}exp(\frac{\lambda }{2}t),\\ {\psi }_{n}^{4} & = & 2{\lambda }^{n+1}exp(\frac{\lambda }{2}t),\end{array}$$

37 $$\{\begin{array}{rcl}{\alpha }_{j}[n] & = & (\frac{1}{{k}_{j}}-1)\lambda ,\\ {\beta }_{j}[n] & = & 4-\frac{2}{{k}_{j}},\\ {\gamma }_{j}[n] & = & (2-\frac{1}{{k}_{j}})\lambda \mathrm{.}\end{array}$$

Substituting Eqs (35) and (36) into Eq. (32), we are easy to get the ${\alpha }_{j}[n]$, ${\beta }_{j}[n]$ and ${\gamma }_{j}[n]$. According to the relations between ${r}_{n},\,{s}_{n},\,{u}_{n},\,{w}_{n}$ and ${\tilde{r}}_{n},\,{\tilde{s}}_{n},\,{\hat{u}}_{n},\,{\tilde{w}}_{n}$ in Eq. (32), the explicit solutions are given rise to as following:

38 $$\begin{array}{l}{\tilde{r}}_{n}=-\,1-\tfrac{({\beta }_{1}[n+1]-1)\,({\beta }_{2}[n+1]-1)\,({\lambda }_{2}-{\lambda }_{1})}{({\alpha }_{1}[n+1]-{\gamma }_{1}[n+1])\,({\beta }_{2}[n+1]-1)-({\alpha }_{2}[n+1]-{\gamma }_{2}[n+1])\,({\beta }_{1}[n+1]-1)},\end{array}$$

39 $$\begin{array}{l}{\tilde{q}}_{n}=-\,\tfrac{({\alpha }_{2}[n]-{\gamma }_{1}[n])\,({\alpha }_{2}[n]-{\gamma }_{2}[n])\,({\lambda }_{2}^{2}-{\lambda }_{1}^{2})}{{\lambda }_{2}({\beta }_{2}[n]-1)\,({\alpha }_{1}[n]-{\gamma }_{1}[n])-{\lambda }_{1}({\beta }_{1}[n]-1)\,({\alpha }_{2}[n]-{\gamma }_{2}[n])},\end{array}$$

40 $$\begin{array}{rcl}{\tilde{\nu }}_{n} & = & 1+\tfrac{1}{2}\tfrac{{\beta }_{1}[n+1](1+{\beta }_{2}[n+1]){\lambda }_{2}-{\beta }_{2}[n+1](1+{\beta }_{1}[n+1]){\lambda }_{1}}{{\gamma }_{1}[n+1]{\beta }_{2}[n+1]-{\gamma }_{2}[n+1]{\beta }_{1}[n+1]}\\  &  & \,\,\,-\,(\tfrac{1}{2})\tfrac{{\alpha }_{1}[n]{\beta }_{2}[n]-{\alpha }_{2}[n]{\beta }_{1}[n]}{{\beta }_{2}[n]{\gamma }_{1}[n]-{\beta }_{1}[n]{\gamma }_{2}[n]}\times \tfrac{({\lambda }_{2}-{\lambda }_{1})\,({\beta }_{1}[n]-1)\,({\beta }_{2}[n]-1)}{({\beta }_{2}[n]-1)\,({\alpha }_{1}[n]-{\gamma }_{1}[n])-({\beta }_{1}[n]-1)\,({\alpha }_{2}[n]-{\gamma }_{2}[n])}\\  &  & \,\,\,-\,(\tfrac{1}{2})\tfrac{({\lambda }_{2}-{\lambda }_{1})\,({\beta }_{1}[n+1]-1)\,({\beta }_{2}[n+1]-1)}{({\beta }_{2}[n+1]-1)\,({\alpha }_{1}[n+1]-{\gamma }_{1}[n+1])-({\beta }_{1}[n+1]-1)\,({\alpha }_{2}[n+1]-{\gamma }_{2}[n+1])}\\  &  & \,\,\,-\,(\tfrac{1}{2})\tfrac{{\beta }_{2}[n+1]-{\beta }_{1}[n+1]}{{\beta }_{2}[n+1]{\gamma }_{1}[n+1]-{\beta }_{1}[n+1]{\gamma }_{2}[n+1]}\\  &  & \,\,\,\times \,\tfrac{({\alpha }_{2}[n+1]-{\gamma }_{2}[n+1])\,({\beta }_{1}[n+1]-1){\lambda }_{1}-({\alpha }_{1}[n+1]-{\gamma }_{1}[n+1])\,({\beta }_{1}[n+1]-1){\lambda }_{2}}{({\beta }_{2}[n+1]-1)\,({\alpha }_{1}[n+1]-{\gamma }_{1}[n+1])-({\beta }_{1}[n+1]-1)\,({\alpha }_{2}[n+1]-{\gamma }_{2}[n+1])},\end{array}$$

41 $$\begin{array}{rcl}{\tilde{w}}_{n} & = & (\tfrac{1}{2})\tfrac{({\alpha }_{2}[n]-{\gamma }_{2}[n])\,({\alpha }_{2}[n]-{\gamma }_{1}[n])\,({\lambda }_{2}^{2}-{\lambda }_{1}^{2})}{{\lambda }_{2}({\beta }_{2}[n]-1)\,({\alpha }_{1}[n]-{\gamma }_{1}[n])-{\lambda }_{1}({\beta }_{1}[n]-1)\,({\alpha }_{2}[n]-{\gamma }_{2}[n])}\\  &  & -\,(\tfrac{1}{2})\tfrac{({\alpha }_{2}[n]+{\beta }_{2}[n]){\gamma }_{2}[n]{\lambda }_{2}-({\alpha }_{1}[n]+{\beta }_{1}[n]){\gamma }_{2}[n]{\lambda }_{1}}{{\beta }_{1}[n]{\gamma }_{2}[n]-{\beta }_{2}[n]{\gamma }_{1}[n]}\\  &  & +\,(\tfrac{1}{2})\tfrac{({\gamma }_{2}[n]-{\gamma }_{1}[n]}{{\beta }_{1}[n]{\gamma }_{2}[n]-{\beta }_{2}[n]{\gamma }_{1}[n]}\times \tfrac{({\alpha }_{2}[n]-{\gamma }_{2}[n])\,({\alpha }_{2}[n]-{\gamma }_{1}[n])\,({\lambda }_{2}^{2}-{\lambda }_{1}^{2})}{{\lambda }_{2}({\beta }_{2}[n]-1)\,({\alpha }_{1}[n]-{\gamma }_{1}[n])-{\lambda }_{1}({\beta }_{1}[n]-1)\,({\alpha }_{2}[n]-{\gamma }_{2}[n])},\\  &  & +\,(\tfrac{1}{2})\tfrac{{\alpha }_{1}[n]{\gamma }_{2}[n]{\lambda }_{2}-{\alpha }_{2}[n]{\gamma }_{1}[n]{\lambda }_{1}}{{\lambda }_{1}{\lambda }_{2}({\beta }_{1}[n]{\gamma }_{2}[n]-{\beta }_{2}[n]{\gamma }_{1}[n])}\\  &  & \times \,\tfrac{{\lambda }_{1}{\lambda }_{2}[{\lambda }_{2}({\alpha }_{2}[n]-{\gamma }_{2}[n])\,({\beta }_{1}[n]-1)-{\lambda }_{1}({\alpha }_{1}[n]-{\gamma }_{1}[n])\,({\beta }_{1}[n]-1)]}{{\lambda }_{2}({\beta }_{2}[n]-1)\,({\alpha }_{1}[n]-{\gamma }_{1}[n])-{\lambda }_{1}({\beta }_{1}[n]-1)\,({\alpha }_{2}[n]-{\gamma }_{2}[n])},\end{array}$$

where ${\beta }_{i}[n+1]$ and ${\gamma }_{i}[n+1]$ are mentioned, and they are obtained through applying DT once again. Starting from the above explicit solitons, we can apply the DT (32) once again, then other soliton solutions of (27) can be obtained. This process can be done continually. Therefore, we can obtain some novel soliton solutions for the coupling lattice equation (27).

**Figure 1:**
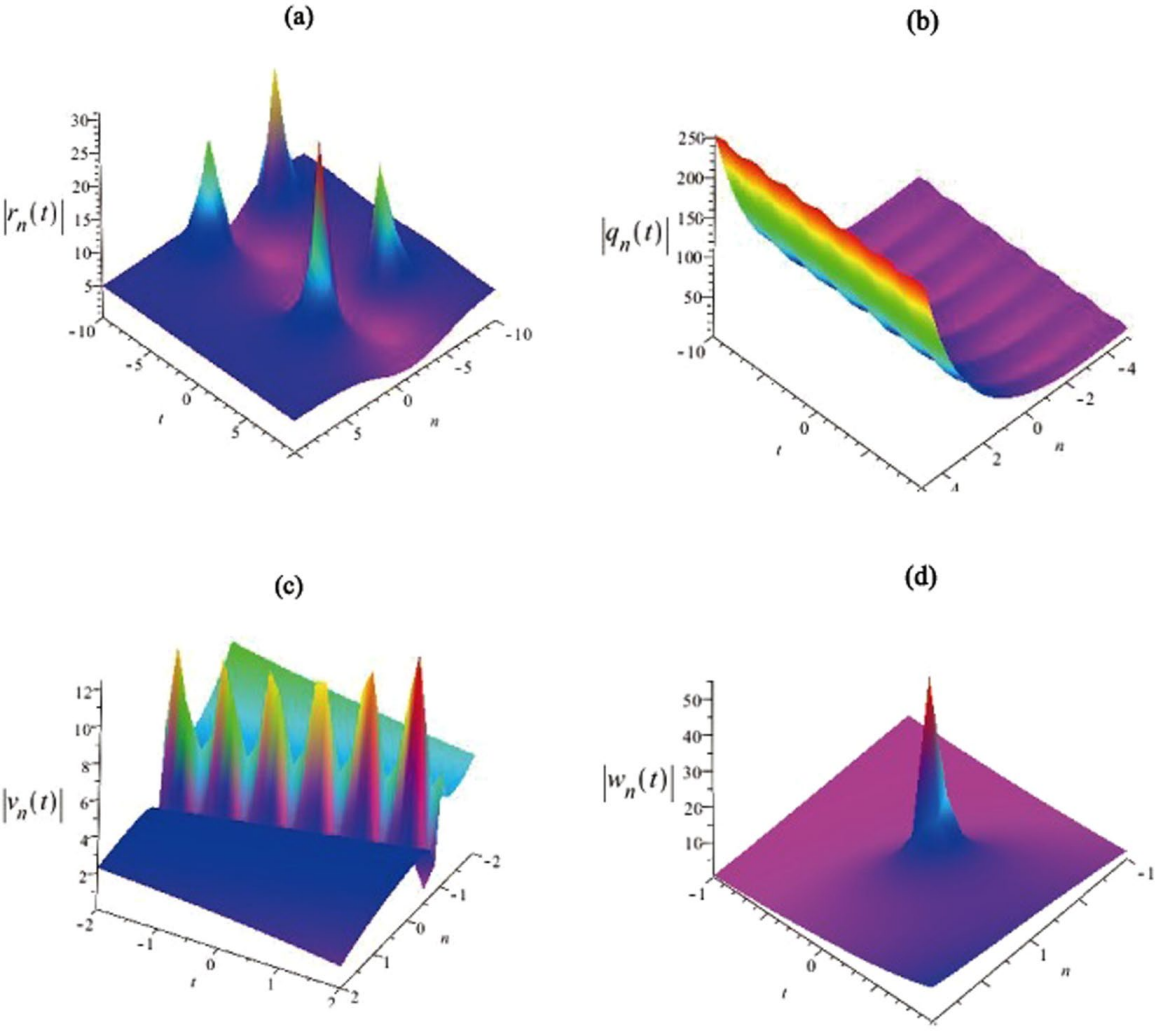
The first kind of intensity distributions for the discrete soliton solutions $|{\tilde{r}}_{n}(t)|,|{\tilde{q}}_{n}(t)|,|{\tilde{v}}_{n}(t)|$ and $|{\tilde{w}}_{n}(t)|$. (**a**) The solution $|{\tilde{r}}_{n}(t)|$ is given by Eq. (38) with the parameters ${\lambda }_{1}=1.5i,{\lambda }_{2}=1.3i,{\lambda }_{3}=2.7i,{\lambda }_{4}=2.4i$, ${k}_{1}=3.8i,{k}_{2}=2.4i$. (**b**) The solution $|{\tilde{q}}_{n}(t)|$ is given by Eq. (39) with the parameters ${\lambda }_{1}=15i,{\lambda }_{2}=13i,{\lambda }_{3}=27i$, ${\lambda }_{4}=24i,{k}_{1}=38i,{k}_{2}=24i$. (**c**) The solution $|{\tilde{v}}_{n}(t)|$ is given by Eq. (40) with the parameters ${\lambda }_{1}=1,\,{\lambda }_{2}=0.5$, ${\lambda }_{3}=0.2,{\lambda }_{4}=0.5,{k}_{1}=1,{k}_{2}=2$. (**d**) The solution $|{\tilde{w}}_{n}(t)|$ is given by Eq. (41) with the parameters ${\lambda }_{1}=3+5i,{\lambda }_{2}=2+4i,{\lambda }_{3}=2+3i,{\lambda }_{4}=6+5i$, ${k}_{1}=1+2i,{k}_{2}=2+4i$.

**Figure 2:**
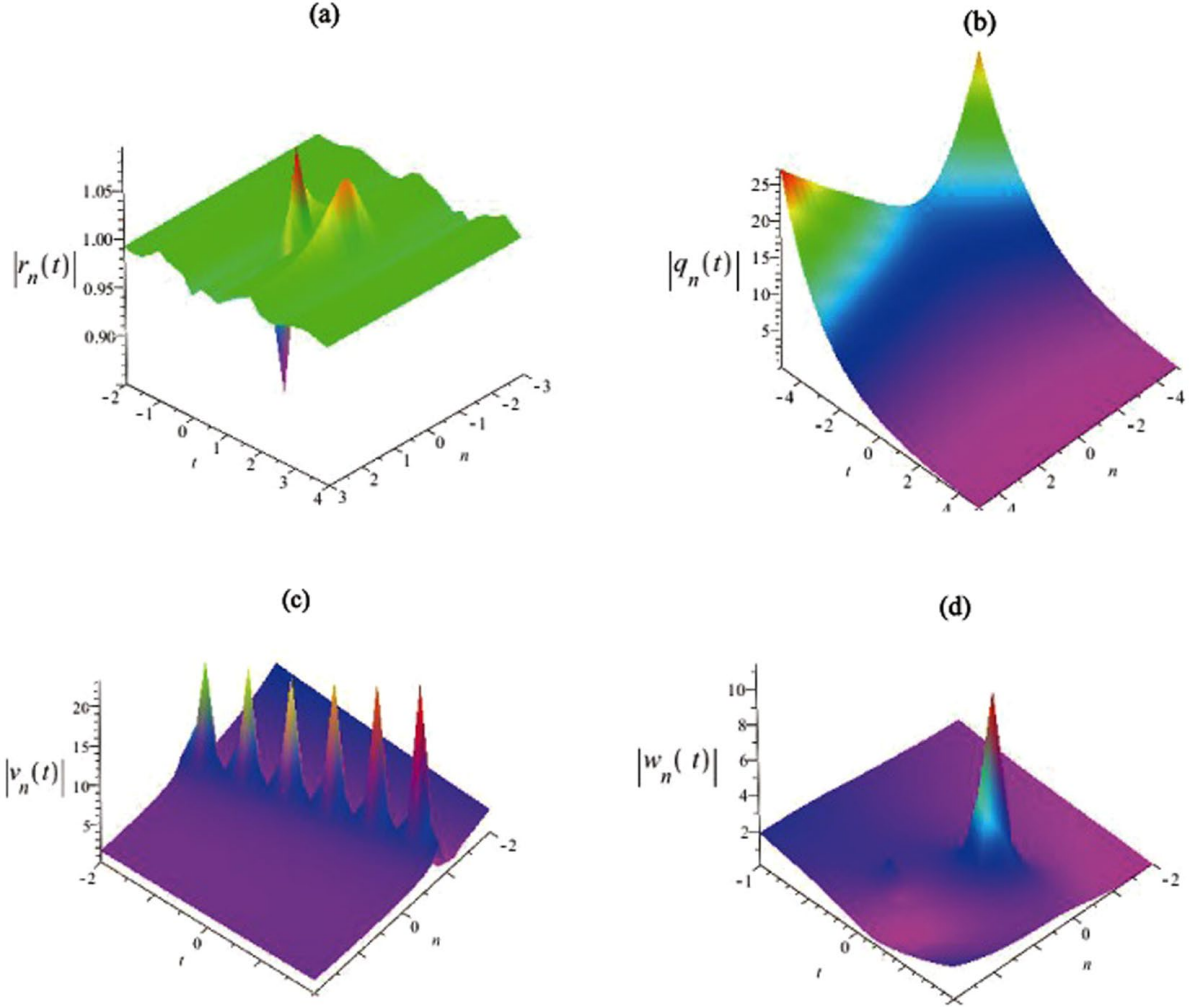
The second kind of intensity distribution for the solutions $|{\tilde{r}}_{n}(t)|,|{\tilde{q}}_{n}(t)|,|{\tilde{v}}_{n}(t)|$ and $|{\tilde{w}}_{n}(t)|$. (**a**) The solution $|{\tilde{r}}_{n}(t)|$ is given by Eq. (38) with the parameters ${\lambda }_{1}=34+5i,{\lambda }_{2}=27+42i,{\lambda }_{3}=20+13i$, ${\lambda }_{4}=26+35i,{k}_{1}=3+5i,{k}_{2}=2+4i$. (**b**) The solution $|{\tilde{q}}_{n}(t)|$ is given by Eq. (39) with the parameters ${\lambda }_{1}=1+1.5i,{\lambda }_{2}=-\,1.3i,{\lambda }_{3}=1.5i,{\lambda }_{4}=-\,2i$, ${k}_{1}=3i,{k}_{2}=-\,2i$. (**c**) The solution $|{\tilde{v}}_{n}(t)|$ is given by Eq. (40) with the parameters ${\lambda }_{1}=1,{\lambda }_{2}=0.5,{\lambda }_{3}=0.2,{\lambda }_{4}=0.5$, ${k}_{1}=0.2,{k}_{2}=12$. (**d**) The solution $|{\tilde{w}}_{n}(t)|$ is given by Eq. (41) with the parameters ${\lambda }_{1}=3+5i,{\lambda }_{2}=4i,{\lambda }_{3}=2+3i,{\lambda }_{4}=5i$, ${k}_{1}=2i,{k}_{2}=2+4i$.

We consider some wave propagations of obtained discrete soliton solutions (38–41) with some free parameters ${\lambda }_{1},{\lambda }_{2},{\lambda }_{3},{\lambda }_{4}$, *k*_1_, *k*_2_ in Figs 1 and 2. Then we give the intensity distributions of the discrete soliton solutions in Eqs (38), (39), (40) and (41) are illustrated. And the evolutions of the discrete soliton solutions given by Eqs (38–41) are shown in Figs 1 and 2. We can find that the amplitude of the discrete soliton invariable with time increases by the parameters ${\lambda }_{1},{\lambda }_{2},{\lambda }_{3},{\lambda }_{4}$ in the single soliton. The soliton velocities of Eqs (38–41) are related to all free parameters presented in this equation. We show the soliton propagations and dynamic evolutions of discrete solutions $|\tilde{r}|,|\tilde{q}|,|\tilde{v}|,|\tilde{w}|$ in Figs 1 and 2.

Based on the DT in Eq. (32), we can obtain the explicit discrete solitons with different parameters in Figs 1 and 2. Figure 1 is similar to the feature of the breather waves, and their figures indicate some sharp compressions and strong amplifications of the non-autonomous soliton solutions. In addition, a special soliton solution is presented in Fig. 2, which has some characters of space-time localized. And it exhibits some features of the rogue waves, but it is based on plane wave background rather than a zero background.

**Figure 3:**
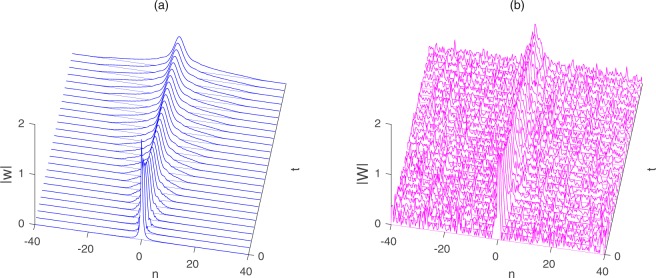
The numerical simulations of intensity distribution for the solution $|{w}_{n}(t)|$. (**a**) Evolution of the amplitude of the solution $|w(n,\,t)|$ (41) with an initial perturbation noise 0.01 and the parameters ${\lambda }_{1}=3+5i$, ${\lambda }_{2}=2+4i,{\lambda }_{3}=2+3i,{\lambda }_{4}=6+5i,{k}_{1}=1+2i,{k}_{2}=2+4i$. (**b**) Evolution of the amplitude of the solution $|w(n,\,t)|$ (41) with an initial perturbation noise 0.17 and the parameters ${\lambda }_{1}=3+5i,\,{\lambda }_{2}=2+4i$, ${\lambda }_{3}=2+3i,{\lambda }_{4}=6+5i,{k}_{1}=1+2i,{k}_{2}=2+4i$.

In these numerical simulations, a pseudo-spectral method in the time domain and a fourth-order Runge-Kutta scheme with an adaptive step-size control in the spatial domain are employed. When the ‘mild’ modulation instability(MI) effect is weak, a rogue wave can be readily observed (Fig. 3). Evolution of the amplitude Fig. 3(a) with a background noise of 0.01 showing the formation of a rogue wave with almost no influence from the background modulation instability. We found that the rogue wave is ‘masked’ in view of the strong modulation instability of the background. To illustrate the situation, the evolutions with maximum MI gains of an initial perturbation noise 0.17 are shown in Fig. 3(b).

We next present our simulation results regarding the bright solitary wave solution for Eq. (40), the initial condition in this code is taken as a single soliton (40) of the generalized coupled Toda equation. Based on some different kinds of parameters, the stability of the solutions is systematically analyzed via numerical simulations with the perturbed noise.

**Figure 4:**
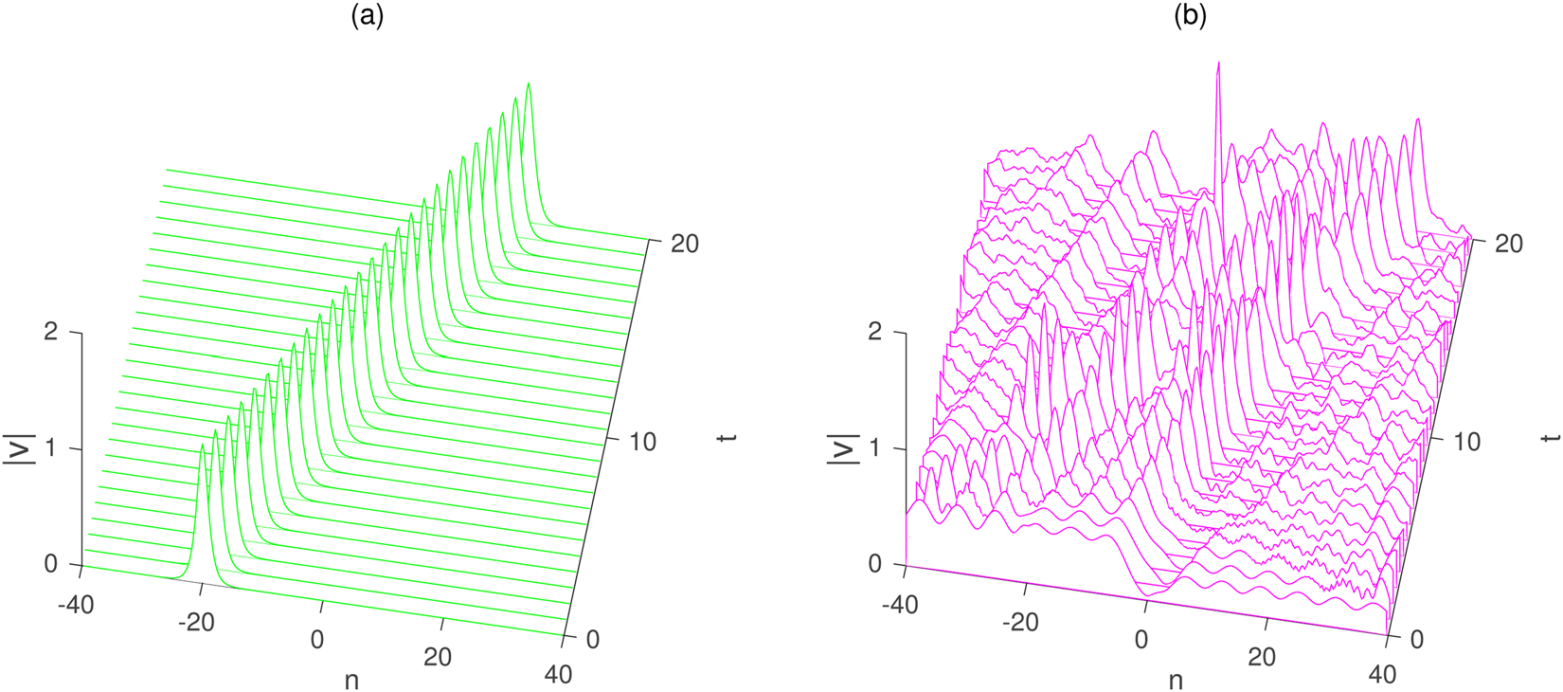
The simulation of bright soliton for the solution $|{v}_{n}(t)|$. (**a**) The solution $|{v}_{n}(t)|$ is given by Eq. (40) with an initial perturbation noise 0.005 and the parameters ${\lambda }_{1}=1,{\lambda }_{2}=0.5,{\lambda }_{3}=0.2,{\lambda }_{4}=0.5$, ${k}_{1}=0.2,{k}_{2}=12$. (**b**) The solution $|{v}_{n}(t)|$ is given by Eq. (40) with an initial perturbation noise 0.1 and the parameters ${\lambda }_{1}=1,\,{\lambda }_{2}=0.5$, ${\lambda }_{3}=0.2,{\lambda }_{4}=0.5,{k}_{1}=0.2,{k}_{2}=12$.

Thus, the stable solution Fig. 4(a) can be very effective to obtain and be easy to be observed in the physical experiments through the numerical simulations. However, we can find that the bright wave propagation (40) is unstable with the perturbed noise 0.1 in Fig. 4(b).

## Discussion

We obtain some novel explicit solutions of the lattice integrable coupling equation with 4 × 4 Lax pairs by a Darboux transformation, and derive the explicit discrete soliton solution, breather solution and rogue wave solution. Then, some properties of these obtained solutions are considered to illustrate the influences of the variable coefficients, which possess the breather and rogue wave soliton structures. Furthermore, we also give some dynamical behaviors of these discrete solutions with the different parameters.

We find that the Darboux transformation(DT) method is very complexed in 4 × 4 matrix spectral problems. And we derive some new solitary solutions with DT method. In the future work, we will take the DT of Lax pairs to apply other discrete integrable coupling equations (the Volterra lattice equation, the discrete Korteweg-de Vried equation and so on). These results might be have some important applications to understanding physical phenomena and experimentations of solitons.

## Methods

### Darboux Transformation for system (27)

In this section, we shall construct the Darboux Transformation for Eq. (27), the Eq. (26) turns into the discrete integrable coupling system in case *m* = 1, which has the following Lax representations:

42 $$E{\phi }_{n}={\phi }_{n+1}={U}_{n}{\phi }_{n}=(\begin{array}{cccc}0 & -{r}_{n} & 0 & {v}_{n}\\ {q}_{n} & \lambda  & {w}_{n} & 0\\ 0 & 0 & 0 & {v}_{n}-{r}_{n}0\\ 0 & 0 & {q}_{n}+{w}_{n} & \lambda \end{array})\,{\phi }_{n},$$

and

43 $${\phi }_{{n}_{t}}={V}_{n}^{1}{\phi }_{n},\,{V}_{n}^{1}=(\begin{array}{cccc}{V}_{n11}^{1} & -{r}_{n-1} & 0 & {v}_{n-1}\\ {q}_{n} & -{V}_{n11}^{1} & {w}_{n} & 0\\ 0 & 0 & -\frac{1}{2}\lambda -{q}_{n}{r}_{n-1} & {v}_{n}-{r}_{n-1}0\\ 0 & 0 & {q}_{n}+{w}_{n} & \frac{1}{2}\lambda +{q}_{n}{r}_{n-1}\end{array}),$$

where ${V}_{n11}^{1}=-\,\frac{1}{2}\lambda +{q}_{n}{r}_{n-1}$. The Lax pairs (42) and (43) are the natural 4 × 4 generalization of the 2 × 2 Lax pairs for the nonlinear equation.

As we known that if a gauge transformation turns the Lax pairs into another Lax pairs of same type, which is called a Darboux transformation. Let’s introduce the gauge transformation of the spectral problems of Eqs (42) and (43).

Firstly, we denote:

44 $${T}_{n}^{N}:\tilde{{\phi }_{n}}={T}_{n}^{N}{\phi }_{n},$$

the ${T}_{n}^{N}$ is of the following form

45 $$(\begin{array}{cccc}{T}_{n11}^{N} & {T}_{n12}^{N} & {T}_{n13}^{N} & {T}_{n14}^{N}\\ {T}_{n21}^{N} & {T}_{n22}^{N} & {T}_{n23}^{N} & {T}_{n24}^{N}\\ 0 & 0 & {T}_{n33}^{N} & {T}_{n34}^{N}\\ 0 & 0 & {T}_{n43}^{N} & {T}_{n44}^{N}\end{array})$$

where

$$\begin{array}{l}{T}_{n11}^{N}={\lambda }^{N}+{{\rm{\Sigma }}}_{i=0}^{N-1}{a}_{n}^{i}{\lambda }^{i},{T}_{n12}^{N}={{\rm{\Sigma }}}_{i=0}^{N-1}{b}_{n}^{i}{\lambda }^{i},\\ {T}_{n13}^{N}={\lambda }^{N}+{{\rm{\Sigma }}}_{i=0}^{N-1}{f}_{n}^{i}{\lambda }^{i},{T}_{n14}^{N}={{\rm{\Sigma }}}_{i=0}^{N-1}{g}_{n}^{i}{\lambda }^{i}\\ {T}_{n21}^{N}={{\rm{\Sigma }}}_{i=0}^{N-1}{c}_{n}^{i}{\lambda }^{i},{T}_{n22}^{N}={\lambda }^{N}+{{\rm{\Sigma }}}_{i=0}^{N-1}{d}_{n}^{i}{\lambda }^{i-1},\\ {T}_{n23}^{N}={{\rm{\Sigma }}}_{i=0}^{N-1}{h}_{n}^{i}{\lambda }^{i},{T}_{n24}^{N}={\lambda }^{N}+{{\rm{\Sigma }}}_{i=0}^{N-1}{L}_{n}^{i}{\lambda }^{i},\\ {T}_{n33}^{N}=2{\lambda }^{N}+{{\rm{\Sigma }}}_{i=0}^{N-1}{a}_{n}^{i}{\lambda }^{i}+{{\rm{\Sigma }}}_{i=0}^{N-1}{f}_{n}^{i}{\lambda }^{i},\\ {T}_{n34}^{N}={{\rm{\Sigma }}}_{i=0}^{N-1}{b}_{n}^{i}{\lambda }^{i}+{{\rm{\Sigma }}}_{i=0}^{N-1}{g}_{n}^{i}{\lambda }^{i},\\ {T}_{n43}^{N}={{\rm{\Sigma }}}_{i=0}^{N-1}{c}_{n}^{i}{\lambda }^{i}+{{\rm{\Sigma }}}_{i=0}^{N-1}{h}_{n}^{i}{\lambda }^{i},\\ {T}_{n44}^{N}=2{\lambda }^{N}+{{\rm{\Sigma }}}_{i=0}^{N-1}{d}_{n}^{i}{\lambda }^{i-1}+{{\rm{\Sigma }}}_{i=0}^{N-1}{L}_{n}^{i}{\lambda }^{i},\end{array}$$

and *N* is a natural number, the ${A}_{n}^{i}$, ${B}_{n}^{i}$, ${C}_{n}^{i}$, ${D}_{n}^{i}$, ${F}_{n}^{i}$, ${G}_{n}^{i}$, ${H}_{n}^{i}$, ${L}_{n}^{i}$ are the functions of *n* and *t*. We have the following relations by Eqs (31), (32) and (44)

46 $$\{\begin{array}{rcl}{\tilde{U}}_{n} & = & {T}_{n+1}^{N}{U}_{n}{({T}_{n}^{N})}^{-1},\\ {\tilde{V}}_{n} & = & ({T}_{{n}_{t}}^{N}+{T}_{n}^{N}{V}_{n})\,{({T}_{n}^{N})}^{-1},\end{array}$$

in which we can proof that the Lax pairs $\tilde{{U}_{n}}$, $\tilde{{V}_{n}}$ have the same forms with *U*_*n*_ and *V*_*n*_.

#### **Proposition 1**.

The Lax pair $\tilde{{U}_{n}}$ defined by (46) is similar to the form of *U*_*n*_,

47 $${\tilde{U}}_{n}=(\begin{array}{cccc}0 & -{\tilde{r}}_{n} & 0 & {\tilde{v}}_{n}\\ {\tilde{q}}_{n} & \lambda  & {\tilde{w}}_{n} & 0\\ 0 & 0 & 0 & {\tilde{v}}_{n}-{\tilde{r}}_{n}0\\ 0 & 0 & {\tilde{q}}_{n}+{\tilde{w}}_{n} & \lambda \end{array}),$$

where the old and new potentials have the following transformation relations:

48 $$\{\begin{array}{rcl}{\tilde{r}}_{n} & = & -{B}_{n+1}^{N-1}+{r}_{n},\\ {\tilde{q}}_{n} & = & -{C}_{n}^{N-1}+{q}_{n},\\ 2{\tilde{v}}_{n} & = & {G}_{n+1}^{N-1}-{B}_{n+1}^{N-1}+2{v}_{n},\\ 2{\tilde{w}}_{n} & = & {C}_{n}^{N-1}-{H}_{n}^{N-1}+2{w}_{n}\mathrm{.}\end{array}$$

#### Proof.

It is easy to find two linear independent solitary solutions ${\phi }_{n}$, ${\psi }_{n}$ about *t* and *λ* from Eq. (31) like

49 $${\phi }_{n}=(\begin{array}{c}{\phi }_{n}^{1}\\ {\phi }_{n}^{2}\\ {\phi }_{n}^{3}\\ {\phi }_{n}^{4}\end{array}),\,{\psi }_{n}=(\begin{array}{c}{\psi }_{n}^{1}\\ {\psi }_{n}^{2}\\ {\psi }_{n}^{3}\\ {\psi }_{n}^{4}\end{array}),$$

and they satisfy the relations ${T}_{n}^{N}{\phi }_{n}={k}_{j}{T}_{n}^{N}{\psi }_{n}$. So the following equations are obtained

50 $$\{\begin{array}{l}\sum _{i=0}^{N-1}\,({A}_{n}^{i}+{\alpha }_{j}[n]{B}_{n}^{i}+{\beta }_{j}[n]{F}_{n}^{i}+{\gamma }_{j}[n]{G}_{n}^{i}){\lambda }_{j}^{i}=-\,(1+{\beta }_{j}[n]){\lambda }_{j}^{N},\\ \sum _{i=0}^{N-1}\,({C}_{n}^{i}+{\alpha }_{j}[n]{D}_{n}^{i}{\lambda }_{j}^{-1}+{\beta }_{j}[n]{H}_{n}^{i}+{\gamma }_{j}[n]{L}_{n}^{i}){\lambda }_{j}^{i}=-\,({\alpha }_{j}[n]+\gamma [n]){\lambda }_{j}^{N},\\ \sum _{i=0}^{N-1}\,[({\beta }_{j}[n]-1){A}_{n}^{i}-({\alpha }_{j}[n]-{\gamma }_{j}[n]){B}_{n}^{i}]{\lambda }_{j}^{i}=-\,({\beta }_{j}[n]-1){\lambda }_{j}^{N},\\ \sum _{i=0}^{N-1}\,({\beta }_{j}[n]-1){C}_{n}^{i}-({\alpha }_{j}[n]-{\gamma }_{j}[n]){D}_{n}^{i}{\lambda }_{j}^{-1}){\lambda }_{j}^{i}=-\,({\alpha }_{j}[n]-\gamma [n]){\lambda }_{j}^{N},\end{array}$$

where $$1\le j\le 2N$$ and

51 $$\{\begin{array}{rcl}{\alpha }_{j}[n] & = & \frac{{\phi }_{n}^{2}-{k}_{j}{\psi }_{n}^{2}}{{\phi }_{n}^{1}-{k}_{j}{\psi }_{n}^{1}},\\ {\beta }_{j}[n] & = & \frac{{\phi }_{n}^{3}-{k}_{j}{\psi }_{n}^{3}}{{\phi }_{n}^{1}-{k}_{j}{\psi }_{n}^{1}},\\ {\gamma }_{j}[n] & = & \frac{{\phi }_{n}^{4}-{k}_{j}{\psi }_{n}^{4}}{{\phi }_{n}^{1}-{k}_{j}{\psi }_{n}^{1}}.\end{array}$$

When choosing *N* = 1, we obtain the values of ${A}_{n}^{0}$, ${B}_{n}^{0}$, ${C}_{n}^{0}$, ${D}_{n}^{0}$, ${F}_{n}^{0}$, ${G}_{n}^{0}$, ${H}_{n}^{0}$, ${L}_{n}^{0}$ by Eqs (50) and (51) as following:

52 $$\{\begin{array}{rcl}{A}_{n}^{0} & = & \tfrac{({\alpha }_{2}[n]-{\gamma }_{2}[n])\,({\beta }_{1}[n]-1){\lambda }_{1}-({\alpha }_{1}[n]-{\gamma }_{1}[n])\,({\beta }_{1}[n]-1){\lambda }_{2}}{({\beta }_{2}[n]-1)\,({\alpha }_{1}[n]-{\gamma }_{1}[n])-({\beta }_{1}[n]-1)\,({\alpha }_{2}[n]-{\gamma }_{2}[n])},\\ {B}_{n}^{0} & = & \tfrac{({\beta }_{1}[n]-1)\,({\beta }_{2}[n]-1)\,({\lambda }_{2}-{\lambda }_{1})}{({\alpha }_{1}[n]-{\gamma }_{1}[n])\,({\beta }_{2}[n]-1)-({\alpha }_{2}[n]-{\gamma }_{2}[n])\,({\beta }_{1}[n]-1)},\\ {C}_{n}^{0} & = & \tfrac{({\alpha }_{2}[n]-{\gamma }_{2}[n])\,({\alpha }_{2}[n]-{\gamma }_{1}[n])\,({\lambda }_{2}^{2}-{\lambda }_{1}^{2})}{{\lambda }_{2}({\beta }_{2}[n]-1)\,({\alpha }_{1}[n]-{\gamma }_{1}[n])-{\lambda }_{1}({\beta }_{1}[n]-1))\,({\alpha }_{2}[n]-{\gamma }_{2}[n])},\\ {D}_{n}^{0} & = & \tfrac{{\lambda }_{1}{\lambda }_{2}[({\alpha }_{2}[n]-{\gamma }_{2}[n])\,({\beta }_{1}[n]-1)-{\lambda }_{1}({\alpha }_{1}[n]-{\gamma }_{1}[n])\,({\beta }_{2}[n]-1)]}{{\lambda }_{2}({\beta }_{2}[n]-1)\,({\alpha }_{1}[n]-{\gamma }_{1}[n])-{\lambda }_{1}({\beta }_{1}[n]-1)\,({\alpha }_{2}[n]-{\gamma }_{2}[n])},\\ {F}_{n}^{0} & = & \tfrac{{\gamma }_{1}[n]({\beta }_{2}[n]+1){\lambda }_{2}-{\gamma }_{2}[n](1+{\beta }_{1}[n]){\lambda }_{1}}{{\gamma }_{2}[n]{\beta }_{1}[n]-{\gamma }_{1}[n]{\beta }_{2}[n]}\tfrac{{\gamma }_{1}-{\gamma }_{2}[n]}{{\beta }_{1}[n]{\gamma }_{2}[n]-{\beta }_{2}[n]{\gamma }_{1}[n]}\\  &  & \times \,\tfrac{({\alpha }_{2}[n]-{\gamma }_{2}[n])\,({\beta }_{1}[n]-1){\lambda }_{1}-({\alpha }_{1}[n]-{\gamma }_{1}[n])\,({\beta }_{1}[n]-1){\lambda }_{2}}{({\beta }_{2}[n]-1)\,({\alpha }_{1}[n]-{\gamma }_{1}[n])-({\beta }_{1}[n]-1)\,({\alpha }_{2}[n]-{\gamma }_{2}[n])}\\  &  & -\,\tfrac{{\alpha }_{1}[n]{\gamma }_{1}[n]-{\alpha }_{2}[n]{\gamma }_{2}[n]}{{\beta }_{1}[n]{\gamma }_{2}[n]-{\beta }_{2}[n]{\gamma }_{1}[n]}\\  &  & \times \,\tfrac{({\lambda }_{2}-{\lambda }_{1})\,({\beta }_{1}[n]-1)\,({\beta }_{2}[n]-1)}{({\beta }_{2}[n]-1)\,({\alpha }_{1}[n]-{\gamma }_{1}[n])-({\beta }_{1}[n]-1)\,({\alpha }_{2}[n]-{\gamma }_{2}[n])},\\ {G}_{n}^{0} & = & \tfrac{{\beta }_{1}[n](1+{\beta }_{2}[n]){\lambda }_{2}-{\beta }_{2}[n](1+{\beta }_{1}[n]){\lambda }_{1}}{{\gamma }_{1}[n]{\beta }_{2}[n]-{\gamma }_{2}[n]{\beta }_{1}[n]}\tfrac{{\beta }_{2}[n]-{\beta }_{1}[n]}{{\gamma }_{1}[n]{\beta }_{2}[n]-{\gamma }_{2}[n]{\beta }_{1}[n]}\\  &  & \times \,\tfrac{({\alpha }_{2}[n]-{\gamma }_{2}[n])\,({\beta }_{1}[n]-1){\lambda }_{1}-({\alpha }_{1}[n]-{\gamma }_{1}[n])\,({\beta }_{1}[n]-1){\lambda }_{2}}{({\beta }_{2}[n]-1)\,({\alpha }_{1}[n]-{\gamma }_{1}[n])-({\beta }_{1}[n]-1)\,({\alpha }_{2}[n]-{\gamma }_{2}[n])}\\  &  & -\,\tfrac{{\alpha }_{1}[n]{\beta }_{2}[n]-{\alpha }_{2}[n]{\beta }_{1}[n]}{{\beta }_{2}[n]{\gamma }_{1}[n]-{\beta }_{1}[n]{\gamma }_{2}[n]}\\  &  & \times \,\tfrac{({\lambda }_{2}-{\lambda }_{1})\,({\beta }_{1}[n]-1)\,({\beta }_{2}[n]-1)}{({\beta }_{2}[n]-1)\,({\alpha }_{1}[n]-{\gamma }_{1}[n])-({\beta }_{1}[n]-1)\,({\alpha }_{2}[n]-{\gamma }_{2}[n])},\\ {H}_{n}^{0} & = & \tfrac{({\alpha }_{2}[n]+{\beta }_{2}[n]){\gamma }_{2}[n]{\lambda }_{2}-({\alpha }_{1}[n]-{\beta }_{1}[n]){\gamma }_{2}[n]{\lambda }_{1}}{{\beta }_{1}[n]{\gamma }_{2}[n]-{\beta }_{2}[n]{\gamma }_{1}[n]}\tfrac{{\gamma }_{2}[n]-{\gamma }_{1}[n]}{{\beta }_{1}[n]{\gamma }_{2}[n]-{\beta }_{2}[n]{\gamma }_{1}[n]}\\  &  & \times \,\tfrac{({\alpha }_{2}[n]-{\gamma }_{2}[n])\,({\alpha }_{2}[n]-{\gamma }_{1}[n])\,({\lambda }_{2}^{2}-{\lambda }_{1}^{2})}{{\lambda }_{2}({\beta }_{2}[n]-1)\,({\alpha }_{1}[n]-{\gamma }_{1}[n])-{\lambda }_{1}({\beta }_{1}[n]-1)\,({\alpha }_{2}[n]-{\gamma }_{2}[n])}\tfrac{{\alpha }_{1}[n]{\gamma }_{2}[n]{\lambda }_{2}-{\alpha }_{2}[n]{\gamma }_{1}[n]{\lambda }_{1}}{{\lambda }_{1}{\lambda }_{2}({\beta }_{1}[n]{\gamma }_{2}[n]-{\beta }_{2}[n]{\gamma }_{1}[n])}\\  &  & \times \,\tfrac{{\lambda }_{1}{\lambda }_{2}[{\lambda }_{2}({\alpha }_{2}[n]-{\gamma }_{2}[n])\,({\beta }_{1}[n]-1)-{\lambda }_{1}({\alpha }_{1}[n]-{\gamma }_{1}[n])\,({\beta }_{2}[n]-1)]}{{\lambda }_{2}({\beta }_{2}[n]-1)\,({\alpha }_{1}[n]-{\gamma }_{1}[n])-{\lambda }_{1}({\beta }_{1}[n]-1)\,({\alpha }_{2}[n]-{\gamma }_{2}[n])},\\ {L}_{n}^{0} & = & \tfrac{{\beta }_{1}[n]({\alpha }_{2}[n]+{\beta }_{2}[n]){\lambda }_{2}-{\beta }_{2}[n]({\alpha }_{1}[n]+{\beta }_{1}[n]){\lambda }_{1}}{{\beta }_{2}[n]{\gamma }_{1}[n]-{\beta }_{1}[n]{\gamma }_{2}[n]}\\  &  & \times \,\tfrac{{\beta }_{2}[n]-{\beta }_{1}[n]}{{\beta }_{2}[n]{\gamma }_{1}[n]-{\beta }_{1}[n]{\gamma }_{2}[n]}\\  &  & \times \,\tfrac{({\alpha }_{2}[n]-{\gamma }_{2}[n])\,({\alpha }_{2}[n]-{\gamma }_{1}[n])\,({\lambda }_{2}^{2}-{\lambda }_{1}^{2})}{{\lambda }_{2}({\beta }_{2}[n]-1)\,({\alpha }_{1}[n]-{\gamma }_{1}[n])-{\lambda }_{1}({\beta }_{1}[n]-1)\,({\alpha }_{2}[n]-{\gamma }_{2}[n])}\\  &  & -\,\tfrac{{\alpha }_{1}[n]{\beta }_{2}[n]{\lambda }_{2}-{\alpha }_{1}[n]{\beta }_{1}[n]{\lambda }_{1}}{{\lambda }_{1}{\lambda }_{2}({\gamma }_{1}[n]{\beta }_{2}[n]-{\gamma }_{2}[n]{\beta }_{1}[n])}\\  &  & \times \,\tfrac{{\lambda }_{1}{\lambda }_{2}[{\lambda }_{2}({\alpha }_{2}[n]-{\gamma }_{2}[n])\,({\beta }_{1}[n]-1)-{\lambda }_{1}({\alpha }_{1}[n]-{\gamma }_{1}[n])\,({\beta }_{2}[n]-1)]}{{\lambda }_{2}({\beta }_{2}[n]-1)\,({\alpha }_{1}[n]-{\gamma }_{1}[n])-{\lambda }_{1}({\beta }_{1}[n]-1)\,({\alpha }_{2}[n]-{\gamma }_{2}[n])}.\end{array}$$

From the above equations, we can know that the ${A}_{n}^{i},\,{B}_{n}^{i},\,{C}_{n}^{i},\,{D}_{n}^{i},\,{F}_{n}^{i},\,{G}_{n}^{i},\,{H}_{n}^{i},\,{L}_{n}^{i}$ are determined by ${\alpha }_{j}[n]$, ${\beta }_{j}[n]$, ${\gamma }_{j}[n]$. In addition, there is the recurrence relations between ${\alpha }_{j}[n+\mathrm{1]}$, ${\beta }_{j}[n+\mathrm{1]}$, ${\gamma }_{j}[n+\mathrm{1]}$ and ${\alpha }_{j}[n]$, ${\beta }_{j}[n]$, ${\gamma }_{j}[n]$. Now we define the following relations

53 $${\alpha }_{j}[n+1]=\frac{{\mu }_{j}[n]}{{\nu }_{j}[n]},\,{\beta }_{j}[n+1]=\frac{{\sigma }_{j}[n]}{{\nu }_{j}[n]},\,{\gamma }_{j}[n+1]=\frac{{\omega }_{j}[n]}{{\nu }_{j}[n]}.$$

And, substituting the Eq. (53) into Eq. (40), we find that the recurrence relations between ${\alpha }_{j}[n+1],{\beta }_{j}[n+1],$ ${\gamma }_{j}[n+1]$ and ${\alpha }_{j}[n],{\beta }_{j}[n],{\gamma }_{j}[n]$ by Eqs (53) and (54) as following

54 $$\{\begin{array}{rcl}{\nu }_{j}[n] & = & -{\gamma }_{n}{\alpha }_{j}[n]+{\nu }_{n}{\gamma }_{j}[n],\\ {\mu }_{j}[n] & = & {q}_{n}+{\lambda }_{j}{\alpha }_{j}[n]+{w}_{n}{\beta }_{j}[n],\\ {\sigma }_{j}[n] & = & ({\nu }_{n}-{r}_{n}){\gamma }_{j}[n],\\ {\omega }_{j}[n] & = & ({q}_{n}-{\omega }_{n}){\beta }_{j}[n]+{\lambda }_{j}{\gamma }_{j}[n].\end{array}$$

When we take Eqs (53) and (54) into Eq. (52), the relations between ${A}_{n+1}^{i}$, ${B}_{n+1}^{i}$, ${C}_{n+1}^{i}$, ${D}_{n+1}^{i}$, ${F}_{n+1}^{i}$, ${G}_{n+1}^{i}$, ${H}_{n+1}^{i}$, ${L}_{n+1}^{i}$ and ${\alpha }_{j}[n],\,{\beta }_{j}[n],\,{\gamma }_{j}[n]$ are easy to be derived. These recurrence relations have vital significance in multi-Darboux transformation.

We assume the ${r}_{n},\,{s}_{n},\,{u}_{n},\,{w}_{n}$ of *U*_*n*_ transform into ${\tilde{r}}_{n},\,{\tilde{s}}_{n},\,{\hat{u}}_{n},\,{\hat{w}}_{n}$ of ${\tilde{U}}_{n}$, two matrix ${\tilde{U}}_{n}$ and *U*_*n*_ are the same structures. Then we look for the relations between the potential functions of ${r}_{n},\,{s}_{n},\,{u}_{n},\,{w}_{n}$ and ${\tilde{r}}_{n},\,{\tilde{s}}_{n},\,{\hat{u}}_{n},\,{\tilde{w}}_{n}$ by ${\tilde{U}}_{n}={T}_{n+1}^{N}{U}_{n}{({T}_{n}^{N})}^{-1}$ in Eq. (46). After careful calculations, we get the following relations:

55 $$-{\tilde{r}}_{n}\,\sum _{i=o}^{N-1}\,{C}_{n}^{i}{\lambda }^{i}={q}_{n}\,\sum _{i=0}^{N-1}\,{b}_{n+1}^{i}{\lambda }^{i},$$

56 $$-{\lambda }^{N}{\tilde{r}}_{n}-{\tilde{r}}_{n}\,\sum _{i=0}^{N-1}\,{d}_{n}^{i}{\lambda }^{i-1}=-\,{r}_{n}{\lambda }^{N}-{r}_{n}\,\sum _{i=0}^{N-1}\,{a}_{n+1}^{i}{\lambda }^{i}+\sum _{i=0}^{N-1}\,{b}_{n+1}^{i}{\lambda }^{i+1},$$

57 $${\lambda }^{N}{\tilde{q}}_{n}+{\tilde{q}}_{n}\,\sum _{i\mathrm{=0}}^{N-1}\,{a}_{n}^{i}{\lambda }^{i}+\sum _{i=o}^{N-1}{c}_{n}^{i}{\lambda }^{i+1}={\lambda }^{N}{q}_{n}+{q}_{n}\sum _{i\mathrm{=0}}^{N-1}{d}_{n+1}^{i}{\lambda }^{i-1},$$

58 $${\tilde{q}}_{n}\sum _{i=0}^{N-1}\,{b}_{n}^{i}{\lambda }^{i}+{\lambda }^{N+1}+\sum _{i=0}^{N-1}\,{d}_{n}^{i}{\lambda }^{i}=-\,{r}_{n}\,\sum _{i=0}^{N-1}\,{C}_{n+1}^{i}{\lambda }^{i}+{\lambda }^{N+1}+\sum _{i=0}^{N-1}\,{d}_{n+1}^{i}{\lambda }^{i},$$

59 $$-{\tilde{r}}_{n}\sum _{i=0}^{N-1}{h}_{n}^{i}{\lambda }^{i}+{\tilde{\nu }}_{n}\,\sum _{i=0}^{N-1}\,{c}_{n}^{i}{\lambda }^{i}+{\tilde{\nu }}_{n}\,\sum _{i=0}^{N-1}\,{h}_{n}^{i}{\lambda }^{i}={q}_{n}\,\sum _{i=0}^{N-1}\,{g}_{n+1}^{i}{\lambda }^{i}+{\omega }_{n}\,\sum _{i=0}^{N-1}\,{b}_{n+1}^{i}{\lambda }^{i}+{\omega }_{n}\,\sum _{i=0}^{N-1}\,{g}_{n+1}^{i}{\lambda }^{i},$$

60 $$\begin{array}{l}-{\tilde{r}}_{n}{\lambda }^{N}-{\tilde{r}}_{n}\,\sum _{i=0}^{N-1}\,{L}_{n}^{i}{\lambda }^{i}+{\tilde{r}}_{n}{\lambda }^{N}+{\tilde{\nu }}_{n}\,\sum _{i=0}^{N-1}\,{d}_{n}^{i}{\lambda }^{i-1}+{\tilde{\nu }}_{n}\,\sum _{i=0}^{N-1}\,{L}_{n}^{i}{\lambda }^{i}\\ \begin{array}{rcl} & = & -{r}_{n}{\lambda }^{N}-{r}_{n}\,\sum _{i=0}^{N-1}\,{f}_{n+1}^{i}{\lambda }^{i}+\sum _{i=0}^{N-1}\,{g}_{n+1}^{i}{\lambda }^{i+1}+{\nu }_{n}{\lambda }^{N}\\  &  & +\,{\nu }_{n}\,\sum _{i=0}^{N-1}\,{d}_{n+1}^{i}{\lambda }^{i}+{\nu }_{n}{\lambda }^{N}\,\sum _{i=0}^{N-1}\,{f}_{n+1}^{i}{\lambda }^{i}+{\nu }_{n}{\lambda }^{N},\end{array}\end{array}$$

61 $$\begin{array}{l}{\tilde{q}}_{n}{\lambda }^{N}+{q}_{n}\,\sum _{i=0}^{N-1}\,{f}_{n}^{i}{\lambda }^{i}+\sum _{i=0}^{N-1}\,{h}_{n}^{i}{\lambda }^{i+1}+{\tilde{\omega }}_{n}{\lambda }^{N}+{\tilde{\omega }}_{n}\,\sum _{i=0}^{N-1}\,{a}_{n}^{i}{\lambda }^{i}+{\tilde{\omega }}_{n}{\lambda }^{N}+{\tilde{\omega }}_{n}\,\sum _{i=0}^{N-1}\,{f}_{n}^{i}{\lambda }^{i}\\ =\,{q}_{n}{\lambda }^{N}+{q}_{n}\,\sum _{i=0}^{N-1}\,{L}_{n+1}^{i}{\lambda }^{i}+{\omega }_{n}{\lambda }^{N}+{\omega }_{n}\,\sum _{i=0}^{N-1}\,{d}_{n+1}^{i}{\lambda }^{i-1}+\,{\omega }_{n}{\lambda }^{N}+{\omega }_{n}\,\sum _{i=0}^{N-1}\,{L}_{n+1}^{i}{\lambda }^{i},\end{array}$$

62 $$\begin{array}{l}{\tilde{q}}_{n}\,\sum _{i=0}^{N-1}\,{g}_{n}^{i}{\lambda }^{i}+{\lambda }^{N+1}+\sum _{i=0}^{N-1}\,{L}_{n}^{i}{\lambda }^{i+1}+{\tilde{\omega }}_{n}\,\sum _{i=0}^{N-1}\,{b}_{n}^{i}{\lambda }^{i}+{\tilde{\omega }}_{n}\,\sum _{i=0}^{N-1}\,{g}_{n}^{i}{\lambda }^{i}\\ =\,-{r}_{n}\,\sum _{i=0}^{N-1}\,{h}_{n+1}^{i}{\lambda }^{i}+{\lambda }^{N+1}+\sum _{i=0}^{N-1}\,{L}_{n+1}^{i}{\lambda }^{i+1}+\,{\nu }_{n}\,\sum _{i=0}^{N-1}\,{c}_{n+1}^{i}{\lambda }^{i}+{\nu }_{n}\,\sum _{i=0}^{N-1}\,{h}_{n+1}^{i}{\lambda }^{i},\end{array}$$

We compare the ranks of *λ*^*N*^ in Eqs (56), (57), (60) and (61), we gain the objective equations as following:

63 $$\{\begin{array}{rcl}{\tilde{r}}_{n} & = & -{B}_{n+1}^{N-1}+{r}_{n},\\ {\tilde{q}}_{n} & = & -{C}_{n}^{N-1}+{q}_{n},\\ 2{\tilde{v}}_{n} & = & {G}_{n+1}^{N-1}-{B}_{n+1}^{N-1}+2{v}_{n},\\ 2{\tilde{w}}_{n} & = & {C}_{n}^{N-1}-{H}_{n}^{N-1}+2{w}_{n}.\end{array}$$

The proof is completed.

#### **Proposition 2**.

Based on the transformation relations (63), the matrix ${\tilde{V}}_{n}$ in (46) has the same form with *V*_*n*_ as following

64 $${\tilde{V}}_{n}=(\begin{array}{cccc}{\tilde{V}}_{n11} & -{\tilde{r}}_{n-1} & 0 & {\tilde{v}}_{n-1}\\ {\tilde{q}}_{n} & -{\tilde{V}}_{n11} & {\tilde{w}}_{n} & 0\\ 0 & 0 & -\frac{1}{2}\lambda -{\tilde{q}}_{n}{\tilde{r}}_{n-1} & {\tilde{v}}_{n}-{\tilde{r}}_{n-1}\\ 0 & 0 & {\tilde{q}}_{n}+{\tilde{w}}_{n} & \frac{1}{2}\lambda +{\tilde{q}}_{n}{\tilde{r}}_{n-1}\end{array}),$$

where

$${\tilde{V}}_{n11}=-\,\frac{1}{2}\lambda +{\tilde{q}}_{n}{\tilde{r}}_{n-1}.$$

#### Proof.

We assume the matrices ${\tilde{V}}_{n}$ and *V*_*n*_ are the same forms, and satisfy the transformation equation of ${\tilde{V}}_{n}=[{({T}_{n}^{N})}_{t}+{T}_{n}^{N}{V}_{n}]\,{({T}_{n}^{N})}^{-1}$ in Eq. (46). We can derive the transformation relations between ${r}_{n},{s}_{n},{u}_{n},{w}_{n}$ of *V*_*n*_ and ${\tilde{r}}_{n},{\tilde{s}}_{n},{\tilde{u}}_{n},{\tilde{w}}_{n}$ of ${\tilde{V}}_{n}$ liking Eq. (63), and prove the gauge transformation under ${T}_{n}^{N}$ can turn the Lax pairs *U*_*n*_, *V*_*n*_ into Lax pairs ${\tilde{U}}_{n},{\tilde{V}}_{n}$ with the same types. In other words, we construct a DT to solve the soliton equation (35). By calculating ${\tilde{V}}_{n}=[{({T}_{n}^{N})}_{t}+{T}_{n}^{N}{V}_{n}]\,{({T}_{n}^{N})}^{-1}$, we have

65 $$\begin{array}{l}-\frac{1}{2}{\lambda }^{N+1}-\frac{1}{2}\,\sum _{i=0}^{N-1}\,{a}_{n}^{i}{\lambda }^{i+1}-{\tilde{r}}_{n+1}{\tilde{q}}_{n}{\lambda }^{N}-{\tilde{r}}_{n-1}{\tilde{q}}_{n}\,\sum _{i=0}^{N-1}\,{a}_{n}^{i}{\lambda }^{i}-{\tilde{r}}_{n}\sum _{i=0}^{N-1}{c}_{n}^{i}{\lambda }^{i}\\ \begin{array}{rcl} & = & -\frac{1}{2}{\lambda }^{N+1}-\frac{1}{2}\,\sum _{i=0}^{N-1}\,{a}_{n}^{i}{\lambda }^{i+1}+\sum _{i=0}^{N-1}\,{d}_{{n}_{t}}^{i}{\lambda }^{i}-{r}_{n-1}{q}_{n}\,\sum _{i=0}^{N-1}\,{a}_{n}^{i}{\lambda }^{i}+{q}_{n}\,\sum _{i=0}^{N-1}\,{b}_{n}^{i}{\lambda }^{i},\end{array}\end{array}$$

66 $$\begin{array}{l}-\frac{1}{2}\,\sum _{i=0}^{N-1}\,{b}_{n}^{i}{\lambda }^{i+1}-{\tilde{r}}_{n}{\tilde{q}}_{n}\,\sum _{i=0}^{N-1}\,{b}_{n}^{i}{\lambda }^{i}-{\tilde{r}}_{n-1}{\lambda }^{N}-{\tilde{r}}_{n-1}\,\sum _{i=0}^{N-1}\,{d}_{n}^{i}{\lambda }^{i-1}\\ \begin{array}{rcl} & = & \sum _{i=0}^{N-1}\,{b}_{n}^{i}{\lambda }^{i}-{r}_{n-1}{\lambda }^{N}-{r}_{n}\,\sum _{i=0}^{N-1}\,{a}_{n}^{i}{\lambda }^{i}+{r}_{n-1}{q}_{n}\,\sum _{i=0}^{N-1}\,{b}_{n}^{i}{\lambda }^{i}+\frac{1}{2}\,\sum _{i=0}^{N-1}\,{b}_{n}^{i}{\lambda }^{i+1},\end{array}\end{array}$$

67 $$\begin{array}{l}{q}_{n}{\lambda }^{N}+{q}_{n}\,\sum _{i=0}^{N-1}\,{a}_{n}^{i}{\lambda }^{i}+\frac{1}{2}\,\sum _{i=0}^{N-1}\,{c}_{n}^{i}{\lambda }^{i+1}+{r}_{n-1}{q}_{n}\,\sum _{i=0}^{N-1}\,{c}_{n}^{i}{\lambda }^{i}\\ \begin{array}{rcl} & = & \sum _{i=0}^{N-1}\,{C}_{{n}_{t}}^{i}{\lambda }^{i}-\frac{1}{2}\,\sum _{i=0}^{N-1}\,{C}_{n}^{i}{\lambda }^{i+1}-{r}_{n-1}{q}_{n}\,\sum _{i=0}^{N-1}\,{C}_{n}^{i}{\lambda }^{i}+{q}_{n}{\lambda }^{N}+{q}_{n}\,\sum _{i=0}^{N-1}\,{d}_{n}^{i}{\lambda }^{i-1},\end{array}\end{array}$$

68 $$\begin{array}{l}{\tilde{q}}_{n}\,\sum _{i=0}^{N-1}\,{b}_{n}^{i}{\lambda }^{i}+\frac{1}{2}{\lambda }^{N+1}+\frac{1}{2}\,\sum _{i=0}^{N-1}\,{d}_{n}^{i}{\lambda }^{i}+{\tilde{r}}_{n-1}{\tilde{q}}_{n-1}{\lambda }^{N}+{\tilde{r}}_{n-1}{\tilde{q}}_{n}\,\sum _{i=0}^{N-1}\,{d}_{n}^{i}{\lambda }^{i-1}\\ \begin{array}{rcl} & = & \sum _{i=0}^{N-1}\,{d}_{{n}_{t}}^{i}{\lambda }^{i-1}-{r}_{n-1}\,\sum _{i=0}^{N-1}\,{c}_{n}^{i}{\lambda }^{i}+\frac{1}{2}{\lambda }^{N+1}+{r}_{n-1}{q}_{n}{\lambda }^{N}\\  &  & +\,\frac{1}{2}\,\sum _{i=0}^{N-1}\,{d}_{n}^{i}{\lambda }^{i}+{r}_{n-1}{q}_{n}\,\sum _{i=0}^{N-1}\,{d}_{n}^{i}{\lambda }^{i-1},\end{array}\end{array}$$

69 $$\begin{array}{c}-\frac{1}{2}{\lambda }^{N+1}-\frac{1}{2}\,\sum _{i=0}^{N-1}\,{f}_{n}^{i}{\lambda }^{i+1}-\,{\tilde{r}}_{n-1}{\tilde{q}}_{n}\,\sum _{i=0}^{N-1}\,{f}_{n}^{i}{\lambda }^{i}-{\tilde{r}}_{n-1}\,\sum _{i=0}^{N-1}\,{h}_{n}^{i}{\lambda }^{i}\\ \begin{array}{ccc}+\,{\tilde{\nu }}_{n-1}\,\sum _{i=0}^{N-1}\,{c}_{n}^{i}{\lambda }^{i}+{\tilde{\nu }}_{n-1}\,\sum _{i=0}^{N-1}\,{h}_{n}^{i}{\lambda }^{i} & = & \sum _{i=0}^{N-1}\,{f}_{n}^{i}{\lambda }^{i}+{w}_{n}\,\sum _{i=0}^{N-1}\,{b}_{n}^{i}{\lambda }^{i}-\frac{1}{2}{\lambda }^{N+1}\end{array}\\ -\,{r}_{n-1}{q}_{n}{\lambda }^{N}-\frac{1}{2}\,\sum _{i=0}^{N-1}\,{f}_{n}^{i}{\lambda }^{i+1}-\,{r}_{n-1}{q}_{n}\,\sum _{i=0}^{N-1}\,{f}_{n}^{i}{\lambda }^{i}+{q}_{n}\,\sum _{i=0}^{N-1}\,{g}_{n}^{i}{\lambda }^{i}+\,{w}_{n}\,\sum _{i=0}^{N-1}\,{g}_{n}^{i}{\lambda }^{i},\end{array}$$

70 $$\begin{array}{c}-\frac{1}{2}\sum _{i=0}^{N-1}{g}_{n}^{i}{\lambda }^{i+1}-{\tilde{r}}_{n-1}{\tilde{q}}_{n}\,\sum _{i=0}^{N-1}\,{g}_{n}^{i}{\lambda }^{i}-\,{\tilde{r}}_{n-1}{\lambda }^{N}-{\tilde{r}}_{n-1}\,\sum _{i=0}^{N-1}\,{l}_{n}^{i}{\lambda }^{i}+{\tilde{V}}_{n}{\lambda }^{N}\\ \begin{array}{rcc}+\,{\tilde{V}}_{n}\,\sum _{i=0}^{N-1}\,{d}_{n}^{i}{\lambda }^{i-1}+{\tilde{V}}_{n-1}{\lambda }^{N}+{\tilde{V}}_{n-1}\,\sum _{i=0}^{N-1}\,{l}_{n}^{i}{\lambda }^{i} & = & \sum _{i=0}^{N-1}\,{g}_{n}^{i}{\lambda }^{i}+{\nu }_{n-1}{\lambda }^{N}+{\nu }_{n-1}\,\sum _{i=0}^{N-1}\,{a}_{n}^{i}{\lambda }^{i}\end{array}\\ -\,{r}_{n-1}{\lambda }^{N}-{\tilde{r}}_{n-1}\,\sum _{i=0}^{N-1}\,{f}_{n}^{i}{\lambda }^{i}+\frac{1}{2}\,\sum _{i=0}^{N-1}\,{g}_{n}^{i}{\lambda }^{i+1}+{r}_{n-1}{q}_{n}\,\sum _{i=0}^{N-1}\,{g}_{n}^{i}{\lambda }^{i}+\,{\nu }_{n-1}{\lambda }^{N}+{r}_{n-1}\,\sum _{i=0}^{N-1}\,{f}_{n}^{i}{\lambda }^{i},\end{array}$$

71 $$\begin{array}{c}{\tilde{q}}_{n}{\lambda }^{N}+{\tilde{q}}_{n}\,\sum _{i=0}^{N-1}\,{f}_{n}^{i}{\lambda }^{i}+\,\frac{1}{2}\,\sum _{i=0}^{N-1}\,{h}_{n}^{i}{\lambda }^{i+1}+{\tilde{r}}_{n-1}{\tilde{q}}_{n}\,\sum _{i=0}^{N-1}\,{h}_{n}^{i}{\lambda }^{i}+{\tilde{\omega }}_{n}{\lambda }^{N}\\ \begin{array}{ccc}+\,{\tilde{\omega }}_{n}\,\sum _{i=0}^{N-1}\,{a}_{n}^{i}{\lambda }^{i}+{\tilde{\omega }}_{n}{\lambda }^{N}+{\tilde{\omega }}_{n}\,\sum _{i=0}^{N-1}\,{f}_{n}^{i}{\lambda }^{i} & = & \sum _{i=0}^{N-1}\,{h}_{n}^{i}{\lambda }^{i}+{\omega }_{n}{\lambda }^{N}+{\omega }_{n}\,\sum _{i=0}^{N-1}\,{d}_{n}^{i}{\lambda }^{i-1}\end{array}\\ -\,\frac{1}{2}\,\sum _{i=0}^{N-1}\,{h}_{n}^{i}{\lambda }^{i+1}-{r}_{n-1}{q}_{n}\,\sum _{i=0}^{N-1}\,{f}_{n}^{i}{\lambda }^{i}+\,{q}_{n}{\lambda }^{N}+{q}_{n}\,\sum _{i=0}^{N-1}\,{l}_{n}^{i}{\lambda }^{i}+{\omega }_{n}{\lambda }^{N}+{\omega }_{n}\,\sum _{i=0}^{N-1}\,{l}_{n}^{i}{\lambda }^{i},\end{array}$$

72 $$\begin{array}{c}{\tilde{q}}_{n}\sum _{i=0}^{N-1}{g}_{n}^{i}{\lambda }^{i}+\frac{1}{2}{\lambda }^{N+1}+\frac{1}{2}\sum _{i=0}^{N-1}{l}_{n}^{i}{\lambda }^{i+1}+\,{\tilde{r}}_{n-1}{\tilde{q}}_{n}{\lambda }^{N}+{\tilde{r}}_{n-1}{\tilde{q}}_{n}\sum _{i=0}^{N-1}{l}_{n}^{i}{\lambda }^{i}\\ \begin{array}{ccccc} &  & +\,{\tilde{w}}_{n}\sum _{i=0}^{N-1}{b}_{n}^{i}{\lambda }^{i}+{\tilde{w}}_{n}\sum _{i=0}^{N-1}{g}_{n}^{i}{\lambda }^{i} & = & \sum _{i=0}^{N-1}\,{l}_{n}^{i}{\lambda }^{i}+{\nu }_{n-1}\,\sum _{i=0}^{N-1}\,{c}_{n}^{i}{\lambda }^{i}\end{array}\\ -\,{r}_{n-1}\,\sum _{i=0}^{N-1}\,{h}_{n}^{i}{\lambda }^{i}+\frac{1}{2}{\lambda }^{N+1}+{r}_{n-1}{q}_{n}{\lambda }^{N}+\,\frac{1}{2}\,\sum _{i=0}^{N-1}\,{l}_{n}^{i}{\lambda }^{i+1}+{r}_{n-1}{q}_{n}\,\sum _{i=0}^{N-1}\,{l}_{n}^{i}{\lambda }^{i}+\,{\omega }_{n-1}\,\sum _{i=0}^{N-1}\,{h}_{n}^{i}{\lambda }^{i}.\end{array}$$

By comparing the ranks of *λ*^*N*+1^ in Eqs (66), (67), (70) and (71), we get the following equations:

73 $$\{\begin{array}{rcl}{\tilde{r}}_{n-1} & = & -{B}_{n}^{N-1}+{r}_{n-1},\\ {\tilde{q}}_{n} & = & -{C}_{n}^{N-1}+{q}_{n},\\ 2{\tilde{\nu }}_{n-1} & = & {G}_{n}^{N-1}-{B}_{n}^{N-1}+2{\nu }_{n-1},\\ 2{\tilde{\omega }}_{n} & = & {C}_{n}^{N-1}-{H}_{n}^{N-1}+2{\omega }_{n}.\end{array}$$

After observing and contrasting the Eqs (63) and (73), we know that they have the same relations between ${r}_{n},\,{s}_{n},\,{u}_{n},\,{w}_{n}$ and ${\tilde{r}}_{n},\,{\tilde{s}}_{n},\,{\hat{u}}_{n},\,{\tilde{w}}_{n}$. That means that we successfully construct a Darboux Transformation ${T}_{n}^{N}$, and the Lax pairs *U*_*n*_, *V*_*n*_ and new Lax pairs ${\tilde{U}}_{n}$, ${\tilde{V}}_{n}$ have the same types with DT method. When *N* = 1 in Eq. (62), we have the relations

74 $$\{\begin{array}{rcl}{\tilde{r}}_{n} & = & -{B}_{n+1}^{0}+{r}_{n},\\ {\tilde{q}}_{n} & = & -{C}_{n}^{0}+{q}_{n},\\ 2{\tilde{\nu }}_{n-1} & = & {G}_{n+1}^{0}-{B}_{n+1}^{0}+2{\nu }_{n-1},\\ 2{\tilde{\omega }}_{n} & = & {C}_{n}^{0}-{H}_{n}^{0}+2{\omega }_{n}.\end{array}$$

The proof is completed.

## References

[1] Blaszak M, Marciniak K (1994). R-matrix approach to lattice integrable systems. J. Math. Phys..

[2] Gordoa PR, Joshi N, Pickering A (2001). On a generalized 2 + 1 dispersive water wave hierarchy. Publ. RIMS (Kyoto)..

[3] Gu, C. H., Li, Y. S. & Tu, G. Z. *Soliton theory and its application*. (Zhejiang Publishing House of Science and Technology, 1990).

[4] Fuchssteiner, B. Coupling of completely integrable systems. (Dordrecht: Kluwer, p125, 1993).

[5] Toda M (1967). Vibration of a chain with nonlinear interaction. J. Phys. Soc. Jpn..

[6] Flaschka H (1974). The Toda lattice. II. Existence of integrals. Phys. Rev. B..

[7] Olive D, Turok N (1985). Local conserved densities and zero-curvature conditions for Toda lattice field theories. Nucl. Phys. B..

[8] Ma WX, Fuchssteiner B (1996). Integrable theory of the perturbation equations. Chaos Solitons Fractals..

[9] Ma WX (2000). Integrable couplings of soliton equations by perturbations I. A general theory and application to the KdV hierarchy. Methods Appl. Anal..

[10] Zhang YF, Fan EG, Zhang YQ (2006). Discrete integrable couplings associated with Toda-type lattice and two hierarchies of discrete soliton equations. Phys. Lett. A..

[11] Xia TC, You FC, Chen DY (2006). A generalized cubic Volterra lattice hierarchy and its integrable couplings system. Chaos, Solitons and Fractals..

[12] Fan EG (2008). A lattice hierarchy and its continuous limits. Phys. Lett. A..

[13] Fan EG, Dai HH (2008). A differential-difference hierarchy associated with relativistic Toda and Volterra hierarchies. Phys. Lett. A..

[14] Zhang YF, Zhang HQ (2002). A direct method for integrable couplings of TD hierarchy. J. Math. Phys..

[15] Yu FJ, Li L (2008). A new method to construct the integrable coupling system for discrete soliton equation with the Kronecker product. Phys. Lett. A..

[16] Ma WX, Xu XX, Zhang YF (2006). Semidirect sums of Lie algebras and discrete integrable couplings. J. Math. Phys..

[17] Ma WX, Chen M (2006). Hamiltonian and quasi-Hamiltonian structures associated with semi-direct sums of Lie algebras. J. Phys. A: Gen. Math..

[18] Ma WX, Zhang Y (2010). Component-trace identities for Hamiltonian structures. Appl. Anal..

[19] Ma WX (2007). A discrete variational identity on semi-direct sums of Lie algebras. J. Phys. A: Math. Theor..

[20] Ma WX, Gao L (2009). Coupling integrable couplings. Modern. Phys. Lett. B..

[21] Zhang YF, Tam HW (2010). Three kinds of coupling integrable couplings of the KdV hierarchy of evolution equations. J Math Phys..

[22] Zhang YF, Tam HW (2010). Four Lie algebras associated to *R*^6^ and their applications. J. Math. Phys..

[23] Zhang YF, Feng BL (2011). A few Lie algebras and their applications for generating integrable hierarchies of evolution types. Commun. Nonl. Scie. Nume. Simu..

[24] Ma WX, Zhu ZN (2010). Constructing nonlinear discrete integrable Hamiltonian couplings. Comp. Math. Appl..

[25] Ma WX (2011). Nonlinear continuous integrable Hamiltonian couplings. Appl. Math. Compu..

[26] Yu FJ (2011). A real nonlinear integrable couplings of continuous soliton hierarchy and its Hamiltonian structure. Phys. Lett. A..

[27] Akhmediev, N. & Ankiewicz, A. *Solitons*: *Nonlinear Pulses and Beams* (Chapman and Hall London, 1997).

[28] Yu FJ (2018). Localized analytical solutions and numerically stabilities of generalized Gross?Pitaevskii (GP(p, q)) equation with speci c external potentials. Appl. Math. Lett..

[29] Barnett MP (2004). Symbolic calculation in chemistry: selected examples. Int. J. Quantum Chem..

[30] Matveev VB, Salle MADarboux (1991). Transformation and Solitons.

[31] Ablowitz MJ, Clarkson PA (1991). Solitons, Nonlinear Evolution Equations and Inverse Scattering.

[32] Wadati M (1975). Wave propagation in nonlinear lattice. I. J. Phys. Soc. Jpn..

[33] Yu FJ, Feng S (2017). Explicit solution and Darboux transformation for a new discrete integrable soliton hierarchy with 44 Lax pairs. Math. Method. Appl. Sci..

[34] Weiss J, Tabor M, Carnevale G (1983). The Painleve property for partial differential equations. J. Math. Phys..

[35] Hirota R (2004). The Direct Method in Soliton Theory.

[36] Deift P, Trubowitz E (1979). Inverse scattering on the line. Comm. Pure and Appl. Math..

[37] Matveev VB, Salle MA (1991). Darboux transformations and solitons.

[38] Gu, C. H., Hu, H. S. & Zhou, Z. X. *Darboux transformations in integrable systems*: *theory and their applications to geometry*. (Springer, 2006).

[39] Terng CL, Uhlenbeck K (2000). Backlund transformations and loop group actions. Comm. Pure Appl. Math..

[40] Novikov, S. P. *et al*. *Theory of solitons*: *the inverse scattering method*. (Springer, 1984).

[41] Yu FJ (2017). Dynamics of nonautonomous discrete rogue wave solutions for an Ablowitz-Musslimani equation with PT-symmetric potential. Chaos..

[42] Yu FJ (2016). Nonautonomous rogue waves and ‘catch’ dynamics for the combined Hirota-LPD equation with variable coefficients. Commun. Nonlinear. Sci. Numer. Simulat..

[43] Ding HY, Xu XX, Zhao XD (2004). A hierarchy of lattice soliton equations and its Darboux transformation. Chin. Phys..

[44] Wu YT, Geng XG (1998). A new hierarchy integrable differential-difference equations and Darboux transformation. J. Phys. A: Math. Gen..

[45] Xu XX (2015). Solving an integrable coupling system of Merola-Ragnisco-Tu lattice equation by Darboux transformation of Lax pair. Commun. Nonlinear. Sci..

[46] Toda M (1989). Theory of Nonlinear Lattices.

[47] Adler M, Moerbeke P (1995). Matrix integrals, Toda symmetries, virasoro constraints, and orthogonal polynomials. Duke Math. J..

[48] Leblond H, Triki H, Sanchez F, Mihalache D (2012). Circularly polarized few-optical-cycle solitons in Kerr media: A complex modified Korteweg- de Vries model. Opt. Commun..

[49] Erbay S, Suhubi ES (1989). Nonlinear wave propagation in micropolar media-I. The general theory. Int. J. Eng. Sci..

[50] Gorbacheva OB, Ostrovsky LA (1983). Nonlinear vector waves in a mechanical model of a molecular chain. Phys. D..

[51] Metiu H, Kitahara K, Ross J (1976). A derivation and comparison of two equations (Landau-Ginzburg and Cahn) for the kinetics of phase transitions. J. Chem. Phys..

[52] Fisher RH (1937). The wave of advance of advantageous gene. Am. Eugen..

[53] Scott AC (1975). The electrophysics of a nerve fiber. Rev. Mod. Phys..

